# *TMEM119* (c.G143A, p.S48L) Mutation Is Involved in Primary Failure of Eruption by Attenuating Glycolysis-Mediated Osteogenesis

**DOI:** 10.3390/ijms25052821

**Published:** 2024-02-29

**Authors:** Mindi Xu, Dandan Wang, Kefan Li, Tianyu Ma, Yixiang Wang, Bin Xia

**Affiliations:** 1 Department of Pediatric Dentistry, Peking University School and Hospital of Stomatology, Haidian District, Beijing 100081, China; xu1@pkuss.bjmu.edu.cn (M.X.); wangdandankq@pkuss.bjmu.edu.cn (D.W.); 1610303104@pku.edu.cn (K.L.); matianyu@stu.pku.edu.cn (T.M.); 2 National Clinical Research Center for Oral Diseases, Peking University School and Hospital of Stomatology, Haidian District, Beijing 100081, China; 3 Central Laboratory, Peking University School and Hospital of Stomatology, Haidian District, Beijing 100081, China

**Keywords:** tooth eruption, eruption disorder, glycolysis, genetics, *TMEM119* gene, osteogenesis

## Abstract

Primary failure of eruption (PFE) is a rare oral disease with an incidence rate of 0.06%. It is characterized by abnormal eruption mechanisms that disrupt tooth eruption. The underlying pathogenic genetic variant and mechanism of PFE remain largely unknown. The purpose of this study was to explore the role of a novel transmembrane protein 119 (*TMEM119*) mutation in two PFE patients in a Chinese family. Information collection was performed on the family with a diagnosis of PFE, and blood samples from patients and healthy family members were extracted. Whole-exome sequencing was performed. Bioinformatics analysis revealed that a heterozygous variant in the *TMEM119* gene (c.G143A, p.S48L) was a disease-associated mutation in this family. Recombinant pcDNA3.1 plasmid-containing wild-type and mutant *TMEM119* expression cassettes were successfully constructed and transfected into MC3T3-E1 cells, respectively. The results of in vitro analysis suggested that the subcellular distribution of the TMEM119 protein was transferred from the cell cytoplasm to the nucleus, and the ability of cells to proliferate and migrate as well as glycolytic and mineralized capacities were reduced after mutation. Furthermore, rescue assays showed that activating transcription factor 4 (ATF4) overexpression rescued the attenuated glycolysis and mineralization ability of cells. Results of in vivo analysis demonstrated that TMEM119 was mainly expressed in the alveolar bone around the mouse molar germs, and the expression level increased with tooth eruption, demonstrated using immunohistochemistry and immunofluorescence. Collectively, the novel *TMEM119* mutation is potentially pathogenic in the PFE family by affecting the glucose metabolism and mineralized function of osteoblasts, including interaction with ATF4. Our findings broaden the gene mutation spectrum of PFE and further elucidate the pathogenic mechanism of PFE.

## 1. Introduction

Tooth eruption refers to the physiological process of teeth moving into the oral cavity through the alveolar bone, which is accomplished under the precise regulation of the dental sac, osteoblasts, osteoclasts and related signal molecules [1,2,3]. At present, there is a lack of understanding of the mechanism that underlies the process of tooth eruption. Existing studies have found that the mechanism of tooth eruption is mainly influenced by the formation of an eruption pathway and the power of tooth eruption. The dental sac significantly contributes to the process of tooth eruption. The crown region of the dental sac can recruit osteoclasts for alveolar bone resorption, and the base of the dental sac participates in alveolar bone formation [4,5,6,7]. In the process of tooth eruption, the motivating force comes from alveolar bone formation at the base of the dental sac during the intra-osseous phase [5,8,9,10,11]. Therefore, the balance of osteogenesis and osteoclasis around the tooth germ is very important for the subsequent smooth eruption of the teeth.

As is widely known, clinically, the pathogenesis of tooth eruption disorder can be local or systemic [12,13], including mucosal scar tissue, gingival fibrous hyperplasia, supernumerary teeth, cysts, tumors, malnutrition, endocrine abnormalities, viral infection, etc. In addition, there is a rare tooth eruption disorder defined as there being no clear local or systemic factors to be found clinically, but the abnormality of the tooth eruption mechanism is called primary failure of eruption (PFE, (OMIM) #125350) [14]. In 1981, for the first time, Proffit and Vig described the main clinical features of PFE [15]. PFE is a rare oral disease with an incidence rate of 0.06% [16]. The typical clinical manifestation is tooth eruption disorder, resulting in open occlusion [10,15,17,18]. Some studies show that PFE has familial aggregation [16,19,20,21] and indicate that the proportion of family cases is almost 85% [14].

According to previous studies, PFE is believed to have a genetic etiology. Mutations in parathyroid hormone receptor type 1 (*PTH1R*) and histone methyltransferase 2C (*KMT2C*) have been considered as the causes linked to PFE [21,22,23,24,25,26,27,28]. Nevertheless, it is unclear whether only *PTH1R* gene mutations cause PFE. Not all individuals carrying *PTH1R* mutations are identified as PFE patients, and not all PFE patients carry mutations in *PTH1R* [5,25,29,30]. The known pathogenic genes in existing studies cannot explain all clinical cases. Therefore, the genetic basis and influence of PFE and the underlying mechanism of the transmembrane protein 119 (*TMEM119*) mutation need to be further studied.

*TMEM119* gene is pleiotropic and plays a role in various physiological and pathological processes. The functions of TMEM119 include but are not limited to the following: (i) TMEM119 is an important molecule regulating bone formation and mineralization [31,32,33,34]. (ii) A single dose of methamphetamine (a potent psychostimulant and sympathomimetic drug) alters the expression of TMEM119, a marker for microglial cells and inflammation [35]. (iii) TMEM119 can promote the proliferation, invasion and migration of various cancer cells, affecting tumor size, clinical stage and overall survival time of patients [36,37,38,39]. (iv) TMEM119 is required for late testicular differentiation in mice [40].

Our study identified a novel mutation in *TMEM119* gene (c.G143A, p.S48L) in a Chinese PFE family. We have researched the relationship between the TMEM119 protein and PFE and further explored the underlying mechanism. Our results expand the gene profile of PFE and further clarify its genetic background, which will help with the early diagnosis of affected individuals with PFE and may lead to appropriate treatment opportunities.

## 2. Results

### 2.1. Clinical Characteristics of Primary Failure of Eruption (PFE) Individuals

**Figure 1:**
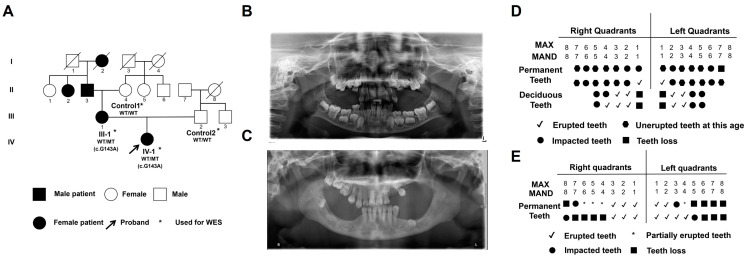
Oral clinical characteristics and analysis of transmembrane protein 119 (TMEM119) mutation in primary failure of eruption (PFE) individuals. (**A**) The pedigree of familial individuals with PFE. The black arrow indicates the proband. Squares and circles represent males and females, respectively. The filled symbols indicate affected individuals. The asterisks indicate individuals who received whole exome sequencing (WES). (**B**) The panoramic radiographs of IV-1 with PFE. (**C**) The panoramic radiographs of III-1 with PFE. (**D**) An illustration of tooth eruption in the situation of IV-1. (**E**) Illustration of tooth eruption in the situation of III-1.

Two individuals from one family with a clinical diagnosis of PFE were recruited, and the family information is shown in Figure 1A. Due to death, illness and lack of personal informed consent, four family members (including two patients and two healthy controls) participated in the study. Oral panoramic radiographs were taken to display the oral phenotypes of two patients (Figure 1B,C). The panoramic X-ray showed that the proband (IV-I, F, 7 years old) had eruption disorder of the deciduous and permanent teeth, despite the fact that the eruption pathway was formed. Her mother (III-1, F, 35 years old) showed multiple missing teeth, insufficient eruption of the upper right posterior teeth and impacted permanent teeth germs in the jaws. The patient reported a history of tooth extraction. The clinical and transmembrane protein 119 (TMEM119) molecular features are summarized in Table 1. The oral phenotype outlined above is in line with classic PFE features, which are characterized as delayed eruption of teeth and posterior open bite (Figure 1D,E).

### 2.2. Identified Mutation and Pathogenic Analysis

**Figure 2:**
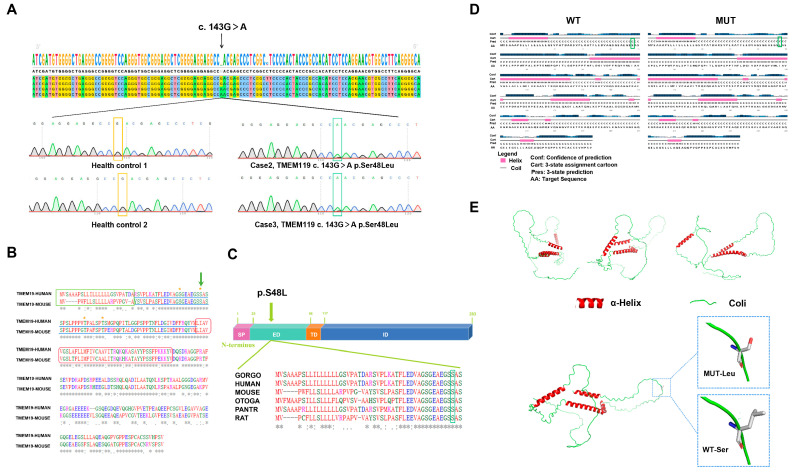
Mutation of transmembrane protein 119 (TMEM119) in primary failure of eruption (PFE) individuals. (**A**) Wild-type and mutant *TMEM119* sequences were analyzed by SnapGene software version 7.0.0 and Chroma software version 2.6.6. The yellow and green boxes indicate the affected site in controls and patients. (**B**) Protein sequences of human and mouse TMEM119 were aligned by UniProt (www.uniprot.org, accessed on 10 August 2022). Blue underline indicates extramembranous domain. Green box indicates N-terminal signal peptides. Red arc box indicates single transmembrane domain. Asterisks indicate potential glycosylation sites. Affected residue is marked with green arrow. (**C**) Diagram of the TMEM119 protein and conservation analysis. SP: signal peptide, ED: extramembranous domain, TD: transmembrane domain, ID: intramembranous domain. Affected residue is marked with green arrow. (**D**) Secondary protein structure analysis of mutated TMEM119. Pink bars represent the helix, and the straight line represents the coil. Transformations have been marked with green boxes. (**E**) Three-dimensional structure model of TMEM119 protein. WT: wild type; MUT: mutant type; Leu: leucine; S: serine.

According to gene function analysis, potential harmfulness prediction, secondary protein structure analysis and conservative analysis, *TMEM119* was selected as a candidate disease gene for the family. This was located in 12q23.3 of the human genome. In order to identify the mutation of the *TMEM119* gene in PFE individuals, we amplified the sequence around the mutant site by PCR, sequenced and blasted through the NCBI website. The results showed that a common mutation was identified in two patients but not in the healthy control. As displayed in Figure 2A, III-1 and IV-1 carried a heterozygous 143 G to A mutation (c.143G > A) in the coding region of *TMEM119* and located in the extra-membranous region, resulting in an amino acid change in Ser48Leu (p.S48L). The amino acid sequence and domain of the TMEM119 protein are displayed in Figure 2B. The results of protein conservation analysis showed that the affected residue in TMEM119 was highly conserved among different species (Figure 2C, green box). Secondary structure analysis showed no change after mutation (Figure 2D, green box). The three-dimensional structure of the TMEM119 protein is displayed in Figure 2E. The amino acid side chain and polarity have changed after gene mutation. As shown in Table S1 (Supplementary Materials), the potential harmfulness prediction implied that the mutation was potentially harmful with SIFT = 0.02 (damaging), PolyPhen-2 = 0.975 (damaging), Mutation Taster = 0.988 (damaging) and Mutation Assessor = 2.075 (medium). The variant in the *TMEM119* gene presented with low allele frequency (Total = 0.0064) from the gnomAD browser (http://gnomad-sg.org/, accessed on 20 January 2022). Therefore, we predicted that the *TMEM119* mutation (c.G143A, p.S48L) may impair the function of the TMEM119 protein and cause PFE. All mutations located in *PTH*, *PTH1R* and *PTHrP* are intergenic, intronic or synonymous exonic variants with a similar expression level among family members, and MAF > 0.01 (Supplementary Figure S1 and Supplementary Table S2).

### 2.3. Overexpression, Subcellular Distribution, Proliferative Activity and Migration Ability Analysis of MC3T3-E1 Cells after TMEM119 Transfection

**Figure 3:**
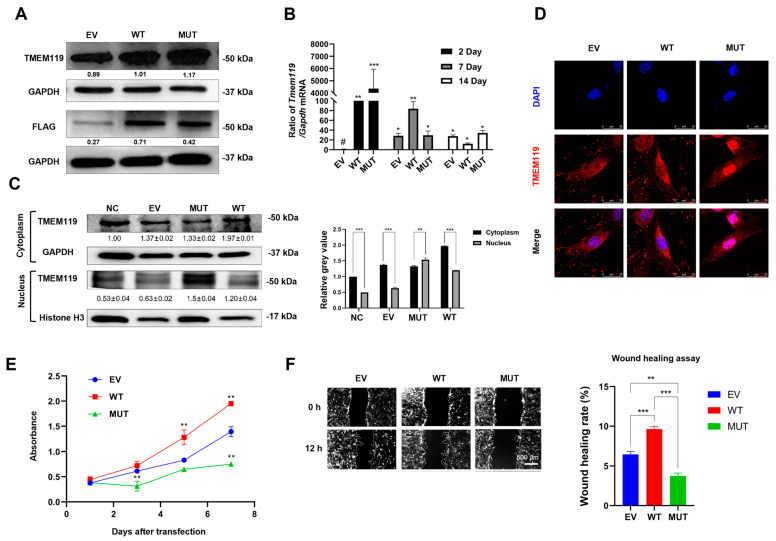
Overexpression, subcellular distribution of transmembrane protein 119 (TMEM119) after transfection and functional analysis of TMEM119 mutation. (**A**) Western blot at 48 h after transfection. (**B**) qPCR results at 2 days, 7 days and 14 days after transfection. # sign represents the control group. (**C**) Nuclear and cytoplasmic protein were separated, and Western blot was performed at 48 h after transfection to analyze subcellular distribution of TMEM119 protein. Relative grey value analysis of Western blot. (**D**) Subcellular localization of TMEM119-FLAG fusion protein in MC3T3-E1 cells. TMEM119 protein was fluorescent red. Nuclei were stained with DAPI (blue). (**E**) Cell Counting Kit-8 assay to analyze cell proliferation ability. (**F**) Wound-healing assay to analyze cell migration ability. Semi-quantitative analysis of wound-healing assay. WT: wild type; MUT: mutant; EV: empty vector. *p* values were significant at * *p* < 0.05, ** *p* < 0.01 and *** *p* < 0.001.

MC3T3-E1 cells were transfected with FLAG-tagged wild-type or mutant TMEM119 plasmid. Western blot analysis revealed that TMEM119 was highly expressed in transfected cells, and both wild-type and mutant TMEM119 generated 50 kDa TMEM119–FLAG fusion protein (Figure 3A). The expression of *TMEM119* mRNA increased for up to 1 week in MC3T3-E1 cells (Figure 3B). PCR products were evaluated by agarose gel electrophoresis (Supplementary Figure S2).

Western blot and semi-quantitative analysis after nuclear and cytoplasmic protein isolation showed that the protein expression was higher in the cytoplasm of WT but higher in nucleus of MUT (Figure 3C). Cell immunofluorescence showed that the TMEM119 fluorescent signals of the control and WT group were localized mainly in the cytoplasm, whereas the mutant TMEM119 protein was mainly distributed in the nucleus (Figure 3D). These results indicated that the subcellular localization of the TMEM119 protein was transferred from the cytoplasm to the nucleus, thus affecting the function of the protein.

The results from CCK-8 suggested that the absorbance levels of cells increased with time. However, the cells of the mutant group were the slowest to proliferate, indicating that the proliferation ability of cells decreased after gene mutation (Figure 3E). A wound-healing assay showed that the wound-healing rate of the EV, WT and MUT group was 6.5%, 9.6% and 3.7%, respectively (Figure 3F). The wound-healing rate of the mutant type was lower than that of the wild group, indicating that the cell migration ability decreased after gene mutation.

### 2.4. TMEM119 Affects Osteogenic Differentiation of MC3T3E1 Cells by Regulating ATF4

**Figure 4:**
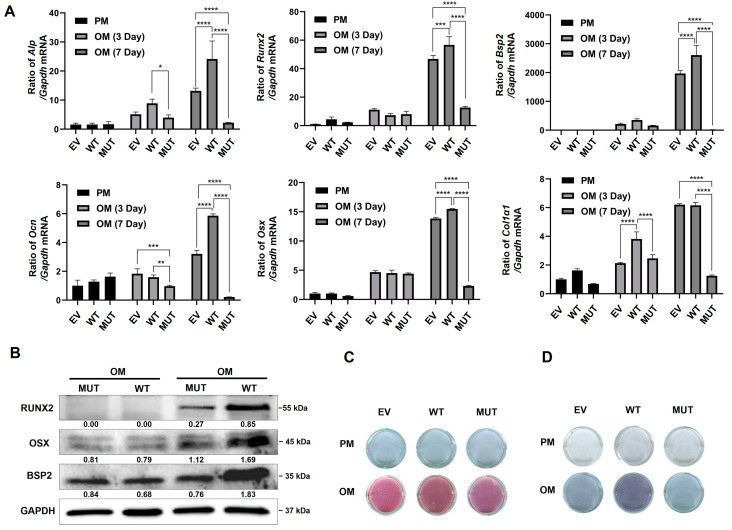
Transmembrane protein 119 (TMEM119) affects osteoblast differentiation. (**A**) The expression of osteoblast biomarkers by qPCR at mRNA levels after transfection and osteogenic induction. (**B**) Western blot analysis of osteoblast protein level after transfection and osteogenic induction. (**C**) Detection of mineralization effect by alkaline phosphatase staining. (**D**) Detection of mineralization effect by alizarin red staining. WT: wild type; MUT: mutant; EV: empty vector. *p* values were significant at * *p* < 0.05, ** *p* < 0.01 and *** *p* < 0.001, **** *p* < 0.0001.

The qPCR results showed that the mRNA levels of osteoblast marker genes were increased with time in all groups. However, the mRNA expression levels in the MUT group were significantly lower than those in the other two groups (Figure 4A).

Western blot analysis showed that the expression of osteogenesis-related biomarkers was down-regulated in the MUT group after osteogenic induction (Figure 4B). ALP staining showed decreased levels of cellular alkaline phosphatase in the MUT group compared with the WT and empty vector groups (Figure 4C). Alizarin red staining showed reduced mineralized nodules in the MUT group compared with the WT and empty vector groups (Figure 4D).

The above results suggest that TMEM119 is an osteoblast-inducing factor that plays an important role in bone formation and normal bone mineralization. Osteogenic differentiation of pre-osteoblasts decreased after *TMEM119* gene mutation.

**Figure 5:**
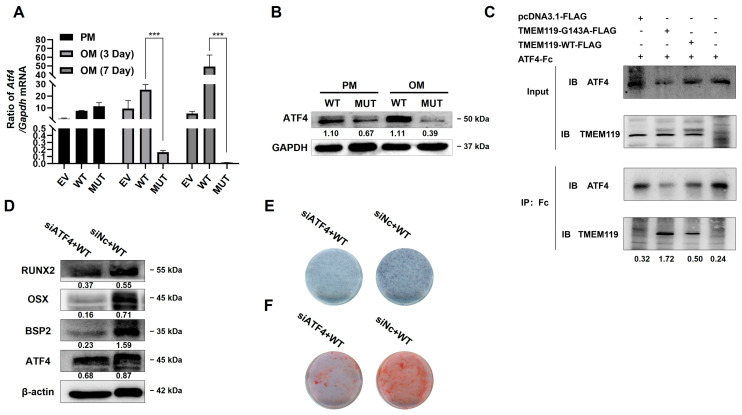
Transmembrane protein 119 (TMEM119) affects osteoblast differentiation by activating transcription factor 4 (ATF4). (**A**) The mRNA expression of ATF4 in different groups after transfection and osteogenic induction. (**B**) Western blot analysis of ATF4 protein after transfection and osteogenic induction. (**C**) Co-immunoprecipitation (Co-IP) analysis of ATF4 and TMEM119-FLAG to detect the interaction between the proteins. (**D**) Western blot analysis of ATF4 and osteogenic protein after knockdown of ATF4 in WT group. (**E**) Detection of mineralization effect by alkaline phosphatase staining after ATF4 knockdown in WT. (**F**) Detection of mineralization effect by alizarin red staining after ATF4 knockdown in WT. WT: wild type; MUT: mutant; EV: empty vector. *p* values were significant at *** *p* < 0.001.

During osteogenic induction, ATF4 expression was increased in WT but decreased in MUT (Figure 5A,B). To further explore the role of ATF4 in the pathway, si-ATF4 was transfected into MC3T3-E1 cells. The qPCR and Western blot results at 2 days after transfection showed that the expression level of ATF4 decreased after knockdown of ATF4 (Supplementary Figure S3).

To explore the interaction between TMEM119 and ATF4, we performed a co-immunoprecipitation (Co-IP) assay. As shown in the results, the IP band of MUT was darker than that of WT, which suggested that the binding between TMEM119 and ATF4 was stronger in the MUT group (Figure 5C).

The siATF4 and plasmid TMEM119-WT were co-transfected into the MC3T3-E1 cells. The results from Western blot analysis showed that the expression of osteogenic biomarkers was down-regulated after ATF4 knockdown (Figure 5D). ALP staining and alizarin red staining showed a decreased level of cellular alkaline phosphatase and reduced mineralized nodules after ATF4 knockdown (Figure 5E,F).

The above results suggest that the interaction between ATF4 and TMEM119 played a positive role in osteogenic differentiation. Osteogenic function was down-regulated after ATF4 knockdown.

### 2.5. Overexpression of ATF4 Rescues TMEM119 Mutation-Caused Osteo-Differentiation Dysfunction and Nuclear Translocation Regulated by TMEM119-ATF4 Interaction in MC3T3-E1 Cells

**Figure 6:**
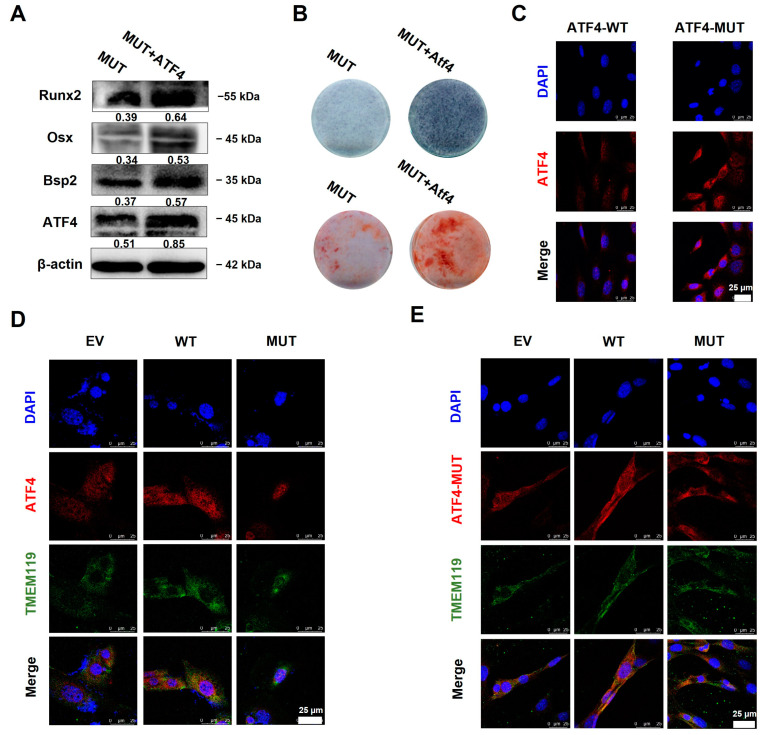
Overexpression of activating transcription factor 4 (ATF4) rescues transmembrane protein 119 (TMEM119) mutation-caused osteo-differentiation dysfunction and nuclear translocation regulated by TMEM119-ATF4 interaction in MC3T3-E1 cells. (**A**) Western blot analysis of ATF4 and osteogenic protein after ATF4 was overexpressed in MUT. (**B**) Detection of mineralization effect by alkaline phosphatase staining and alizarin red staining after ATF4 was overexpressed in MUT. (**C**) Subcellular localization of ATF4 protein. ATF4 was localized in the nucleus, and ATF4 with nuclear localization sequence knocked out (ATF4-MUT) was translocated into the cytoplasm. (**D**) Subcellular localization of ATF4 (red) and TMEM119 (green) protein after co-transfection. Nuclei were stained with DAPI (blue). (**E**) Subcellular localization of TMEM119 (green) and ATF4 (red) protein without nuclear localization sequence. Nuclei were stained with DAPI (blue). WT: wild type; MUT: mutant; EV: empty vector.

The plasmids ATF4 and TMEM119-MUT were co-transfected into the MC3T3-E1 cells. The Western blot assay showed that the expression of osteogenic biomarkers was up-regulated after ATF4 was overexpressed (Figure 6A). ALP staining and alizarin red staining showed increased levels of cellular alkaline phosphatase and mineralized nodules after ATF4 was overexpressed in the MUT group (Figure 6B).

ATF4 and TMEM119 plasmids were co-transfected into the MC3T3-E1 cells and cultured for 2 days. The cell immunofluorescence results showed that in the WT group, ATF4 was located in the nucleus, and TMEM119 was mainly located in the cytoplasm. ATF4 with nuclear localization sequence knocked out (ATF4-MUT) was translocated into the cytoplasm (Figure 6C). However, TMEM119 was partially translocated into the nucleus after the gene mutation (Figure 6D). Subsequently, an ATF4-MUT plasmid was constructed and then co-transfected with TMEM119 plasmid. The cell immunofluorescence results showed that ATF4-MUT was translocated into the cytoplasm. Meanwhile, the mutant TMEM119 also failed to enter the nucleus (Figure 6E).

The above results suggest that overexpression of ATF4 rescued TMEM119 mutation-caused osteogenic differentiation in MC3T3-E1 cells. The interaction between ATF4 and TMEM119 plays a vital part in the nuclear entry of mutant TMEM119.

### 2.6. Decrease in Glycolysis Function Impacted by Reduced Expression of ATF4 Monitored by Seahorse Assay

**Figure 7:**
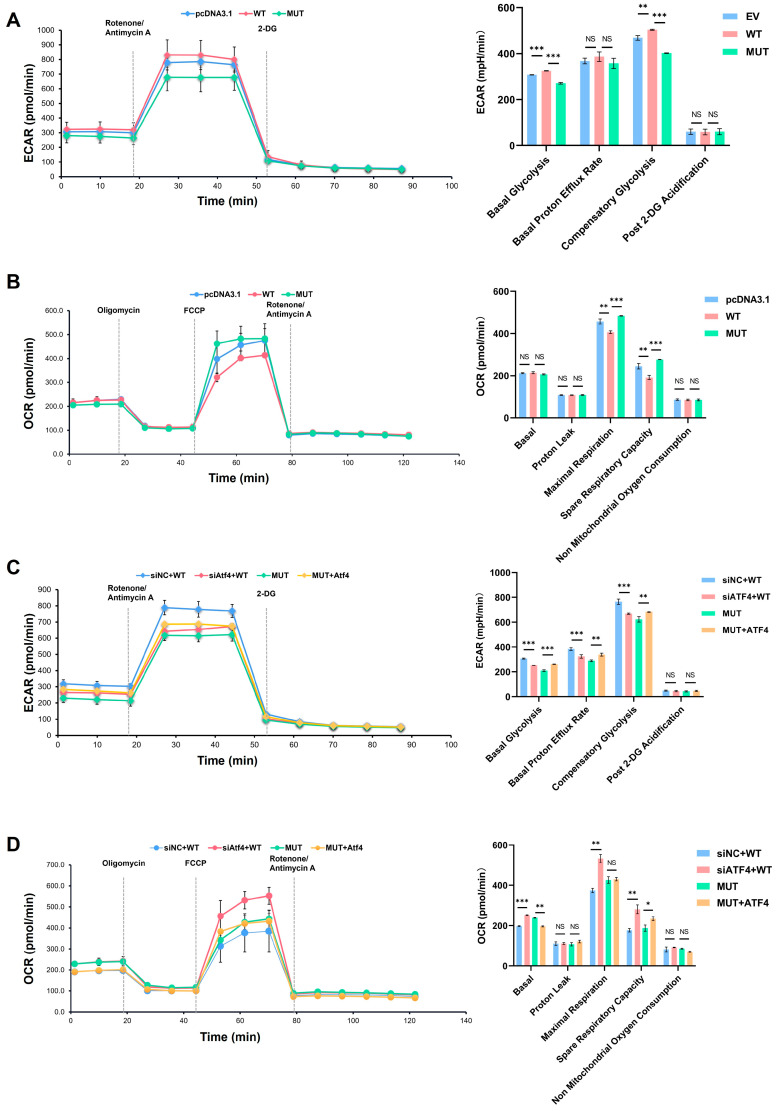
Real-time monitoring of cellular glucose metabolism. (**A**) Glycolytic function assay in pcDNA3.1, WT and MUT groups. (**B**) Respiratory function assay in pcDNA3.1, WT and MUT groups. (**C**) Glycolytic function assay after activating transcription factor 4 (ATF4) overexpression in MUT or knockdown in WT. (**D**) Respiratory function assay after ATF4 overexpression in MUT or knockdown in WT. WT: wild type; MUT: mutant. *p* values were significant at * *p* < 0.05, ** *p* < 0.01 and *** *p* < 0.001, NS indicated no statistical difference.

A glycolysis rate assay showed that the basal and compensatory glycolysis of the cells was weakened in MUT compared with the WT and empty vector groups (Figure 7A). After the knockdown of ATF4 by siATF4 in the WT group, basal and compensatory glycolysis was weakened. However, overexpression of ATF4 in the MUT group enhanced the glycolysis function (Figure 7C).

The results from the mitochondrial stress test showed that the oxidative phosphorylation of cells in the basal state was enhanced after the gene mutation (Figure 7B). After the knockdown of ATF4 in MC3T3-E1 cells with wild-type TMEM119, the cells had enhanced oxidative phosphorylation and spare respiratory capacity in basal and stressed states. However, overexpression of ATF4 in the MUT TMEM119 group led to a decline in the oxidative phosphorylation of cells, and the spare respiratory capacity was also weakened (Figure 7D).

### 2.7. TMEM119 Mutation Inhibits Osteogenic Differentiation of MC3T3-E1 through ATF4-Mediated Glycolysis

**Figure 8:**
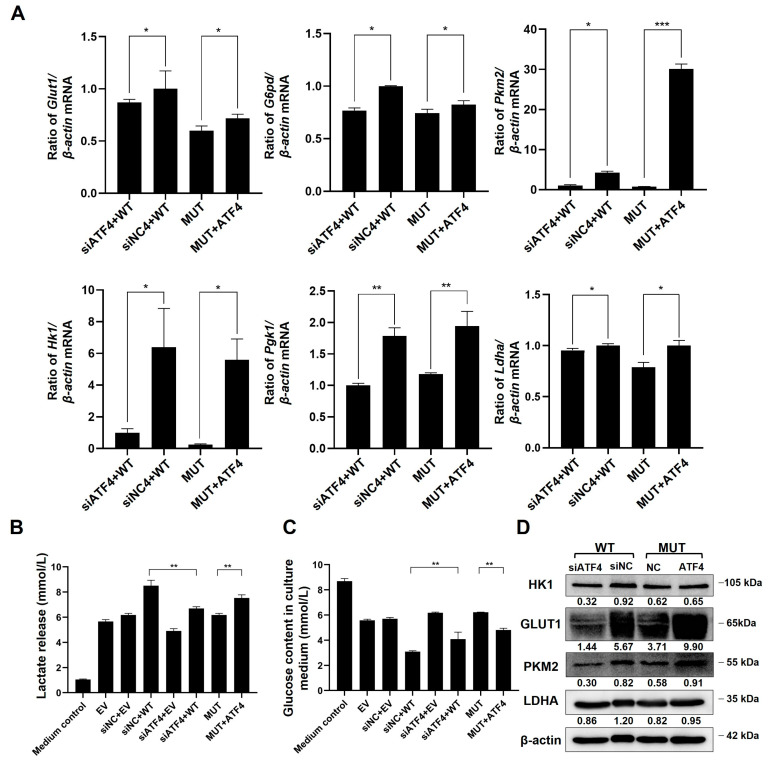
Transmembrane protein 119 (TMEM119) mutation inhibits osteogenic differentiation of MC3T3-E1 by activating transcription factor 4 (ATF4) down-regulation-mediated glycolysis. (**A**) mRNA levels of glycolytic biomarkers after ATF4 overexpression in MUT or knockdown in WT. (**B**) Measurement of glucose level in culture medium. (**C**) Measurement of lactate level in culture medium. (**D**) Protein expression of glycolytic markers after ATF4 overexpression in MUT or knockdown in WT. WT: wild type; MUT: mutant. *p* values were significant at * *p* < 0.05, ** *p* < 0.01 and *** *p* < 0.001.

The qPCR results showed that the level of glycolysis-related genes was reduced after *TMEM119* gene mutation. These genes were also down-regulated in the WT group after ATF4 knockdown. However, after overexpression of ATF4 in MUT, these genes were up-regulated (Figure 8A). Glucose consumption and acid production in the medium of the WT group were reduced, and glycolysis was weakened after knockdown of ATF4. However, overexpression of ATF4 resulted in increased glucose consumption and acid production in MUT, suggesting the enhanced glycolysis of cells (Figure 8B,C). The results of Western blot analysis were consistent with qPCR results (Figure 8D).

The above results demonstrate that after TMEM119 mutation, the glycolytic function of cells and the osteogenic function were weakened, and the oxidative phosphorylation function was enhanced. Meanwhile, TMEM119 was again shown to be an important molecule in cellular glycolysis impacted by ATF4.

### 2.8. Expression of TMEM119 around Mouse Tooth Germ during Tooth Development

**Figure 9:**
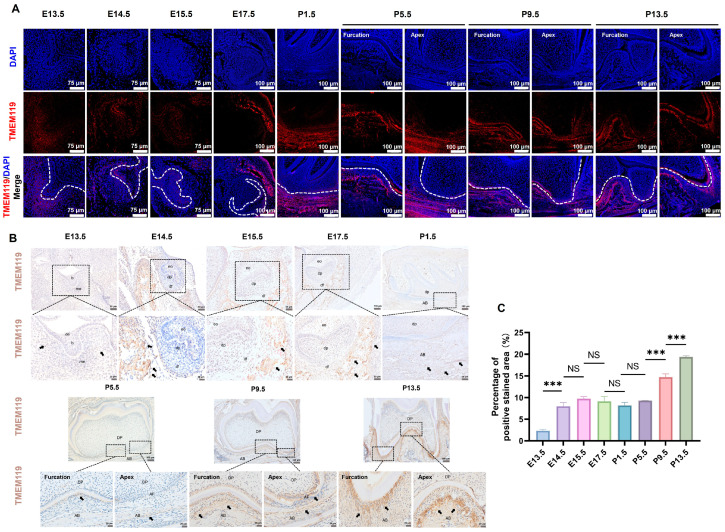
Expression of transmembrane protein 119 (TMEM119) around mice tooth germ during tooth development. (**A**) Immunofluorescence showed TMEM119 protein (red) expression around the tooth germ. Nuclei were stained with DAPI (blue). (**B**) Immunohistochemistry staining (original magnification, 100×/200×/400×) showed TMEM119-positive area around the tooth germ was stained brown. Black arrows indicate positive staining sites. (**C**) Semi-quantitative analysis of immunohistochemical staining. *p* values were significant at *** *p* < 0.001, NS indicated no statistical difference.

The results of immunofluorescence showed that during the development of the mouse tooth germ, a fluorescent signal was visible in the jaw around the tooth germ, root furcation and periapical area (Figure 9A). Similarly, immunohistochemical results showed that TMEM119-positive staining can be seen in the jaw adjacent to the tooth germ, root furcation and periapical area, and positive staining is gradually strengthened during tooth eruption (Figure 9B). Positive and negative control groups of immunohistochemical staining are shown in Supplementary Figure S4. The results of semi-quantitative analysis showed that the TMEM119 expression level had no obvious change in the early stage (E14.5-P5.5) of tooth germ development. With the formation of the root and tooth eruption (P5.5-P13.5), the TMEM119 expression level significantly increased (Figure 9C).

The above results clarify that TMEM119 is highly expressed around the root of the tooth germ, which promotes bone formation and becomes an eruptive force for the tooth.

## 3. Discussion

We presented a new primary failure of eruption (PFE)-related gene mutation, *TMEM119* (c.143G > A, p.48S > L), whose relationship with PFE had not been previously reported. In vitro experiments confirmed that the protein subcellular localization change affected the glycolysis function of osteoblasts by down-regulating the expression of ATF4 after TMEM119 mutation, thus affecting the osteogenic differentiation of osteoblasts. These results elucidated the pathogenesis of PFE from the perspective of cell energy metabolism. Through rescue experiments, we found that overexpression of ATF4 could reverse the osteoblast dysfunction caused by the TMEM119 mutation.

We included a PFE family, wherein two patients who underwent clinical examination showed typical features of PFE. The clinical characteristics show that there are extensive differences among individuals in the same family, indicating the diversity of clinical manifestations of PFE, which is consistent with previous studies [18,25]. This may be caused by a variety of genetic backgrounds or genetic polymorphisms. We also found that individuals carrying the same mutation in the same family have different phenotypes; this may be related to gene expressivity and penetrance. Therefore, the gene–phenotype relationship of PFE needs further study.

Through bioinformatics analysis, we described a common *TMEM119* gene mutation (c.143G > A, p.48S > L) in the two PFE patients described above. To the best of our knowledge, this mutation had not been reported before, nor had there been any report on the relationship between the TMEM119 mutation and PFE. Tooth eruption depends on the formation of alveolar bone at the root region [5,30]. TMEM119 is an important molecule regulating bone formation and mineralization [31,32,33,40]. Bone formation and mineralization were significantly reduced in *Tmem119*^−/−^ mice [31]. In vitro studies showed that in MC3T3-E1 cells, overexpression of TMEM119 promoted osteoblast differentiation, and knockdown of TMEM119 inhibited osteogenic differentiation. In our in vivo experiments, we observed that during the development and eruption of mice teeth, the TMEM expression around the tooth germ was gradually enhanced. Therefore, we considered that osteoblast dysfunction surrounding the tooth germ may be one of the causes of PFE. In view of the close relationship between TMEM119 and osteogenesis and the results of bioinformatics analysis, we predicted *TMEM119* as a candidate pathogenic gene in this family. Previous studies have shown that the TMEM119 protein is a single-transmembrane protein, which is highly conserved among species and has a high degree of homology between humans and mice [41]. The protein is located in the endoplasmic reticulum membrane and functions in the form of a ligand. The extra-membrane area is the main functional area for protein interaction and promoting osteogenesis. The results in this study showed that the mutation was in the extra-membrane region of TMEM119, resulting in the conversion of the 48th amino acid from serine to leucine. Gene mutation may lead to changes in amino acid polarity and glycosylation modifications, which affect protein localization, function and protein–protein interactions.

To further explore the potential relationship between the functional changes of this gene mutation and PFE, we conducted related experiments. Our results indicated that the subcellular localization of the protein had changed after TMEM119 mutation, translocating from the cytoplasm to the nucleus. Thus, after TMEM119 mutation, the proliferation and migration ability of cells and the osteogenic differentiation function were decreased. Different stages of osteoblast differentiation and function require energy participation, and abnormal energy metabolism will lead to abnormal function. Previous studies have shown that osteoblasts mainly rely on glycolysis for energy, with lactic acid as the main product [42,43]. Moreover, glycolysis is enhanced with osteogenic differentiation [43,44]. Meanwhile, an appropriate level of endoplasmic reticulum stress promotes osteogenic differentiation and inhibits cell apoptosis. Activating transcription factor 4 (ATF4) encodes a transcription factor and has been shown to be an important regulator in osteogenic differentiation, promoting the expression of osteogenic-related biomarkers and enhancing aerobic glycolysis [33,45,46,47].

Based on the above results, we hypothesized that ATF4 could play a crucial role in the mechanism through which the TMEM119 mutation induces decreased osteogenic function resulting from decreased glycolysis function. In line with our hypothesis, our results showed that the ATF4 expression level was decreased, glycolysis was weakened, oxidative phosphorylation was enhanced, and osteogenic differentiation was weakened after gene mutation. After ATF4 knockdown in wild-type cells, the glycolysis and osteogenic differentiation of cells were weakened. The above results indicated that the TMEM119 mutation could down-regulate the expression of osteogenic differentiation and glycolysis-related biomarkers through ATF4, resulting in the weakening of cell mineralization ability.

Therefore, we suggested that ATF4 could be a key target for the regulation or amelioration of the decreased osteoblastic function of cells caused by TMEM119 mutations. Our results showed that when the mutant cells overexpressed ATF4, the localization of the mutant TMEM119 protein in cells was corrected to a certain extent. At the same time, the glycolysis level and osteogenic differentiation function of the cells were also partially compensated to some extent. Therefore, we believe that ATF4 can be used as a target to improve osteogenic function regulated by TMEM119.

Wild ATF4 (ATF4-WT) is mainly expressed in the nucleus. Although the expression level of ATF4 in cells decreased after gene mutation, its binding force with TMEM119 was enhanced, and ATF4 carried TMEM119 into the nucleus. After the nuclear localization sequence of ATF4 was knocked out (ATF4-MUT), its expression was transferred to the cytoplasm, and TMEM119 could not enter the nucleus, further proving that ATF4 plays a vital part in the nuclear entry of TMEM119.

However, the roles of TMEM119 and its related molecular networks during the process of tooth eruption need to be further elucidated. PFE is a complex tooth eruption disorder, and it is evident that the role of abnormal periodontal membrane structure, root development and local blood circulation in PFE requires further study. Whether there are other potential pathogenic genes and related mechanisms of PFE remains to be discovered. Due to sample size limitation, we have so far discovered the TMEM119 mutation in this particular family. The genetic background of more PFE patients should be studied in the future for broader implications generally in PFE cases.

**Figure 10:**
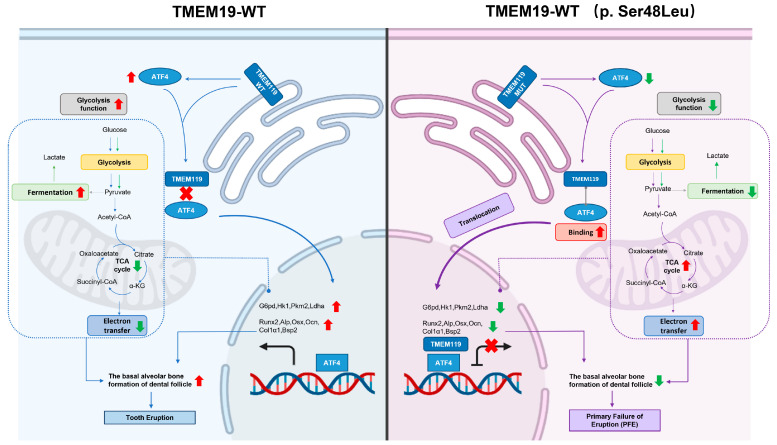
Schematic illustration delineating the role of transmembrane protein 119 (TMEM119) mutation in PFE. In the process of pre-osteoblast cell differentiation, TMEM119 and activating transcription factor 4 (ATF4) play a pivotal role. The reduced expression of ATF4 leads to decreased osteogenesis because of the TMEM119 mutation (p. Ser48Leu) differing from wild-type TMEM119. TMEM119 is translocated into the nucleus due to TMEM119–ATF4 interaction, thus affecting the glucose metabolism and mineralization function of osteoblasts by regulating ATF4. The osteogenic function of the cells is affected, resulting in reduced mineral deposition at the base of the dental sac, which may lead to tooth eruption disorder in primary failure of eruption.

In conclusion, we describe a *TMEM119* mutation (c.143G > A, p.48S > L) in a PFE family, propose for the first time a potential association of TMEM119 and PFE and broaden the genetic profile of PFE. Functional experiments confirmed that the mutation affects the osteogenic and glycolytic function through the interaction of TMEM119 and ATF4. ATF4 overexpression can rescue dysfunction caused by the TMEM119 mutation. We propose a mechanism scheme of TMEM119 regulating PFE in Figure 10.

## 4. Materials and Methods

### 4.1. Recruitment and Clinical Examination of Primary Failure of Eruption (PFE) Individuals

The study was approved by the Ethics Committee of Peking University School and the Hospital of Stomatology (21 June 2019, PKUSSIRB-201946077). Informed consent was obtained by all participants recruited to the study. Two patients with a clinical diagnosis of PFE and two healthy family members were recruited to the study. Clinical examinations were performed, and radiographic images were collected.

### 4.2. Whole-Exome Sequencing (WES) and Mutation Analysis

Peripheral blood samples from the PFE patients and normal individuals were collected for DNA extraction using the TIANamp Genomic DNA Kit (TIANGEN, Beijing, China) according to the manufacturer’s instructions. Whole-genome exome sequencing and analysis was carried out. Calling analysis, alignment analysis and quality control of the WES data can be found in Supplementary Tables S3 and S4. Sequences were displayed and analyzed by SnapGene software version 7.0.0 (NIH, Bethesda, MD, USA) and Chromas software version 2.6.6 (Technelysium, South Brisbane, Australia). Assessing the risk score of the potential harmful genes, we identified candidate genes. The priority of candidate genes was sequenced using ToppGene (https://toppgene.cchmc.org/prioritization.jsp, accessed on 5 February 2022). Training genes, test genes and test results are shown in Supplementary Tables S5 and S6. The candidate genes were reconfirmed by polymerase chain reaction (PCR) and DNA sequencing. More details of the process for mutation screening and pathogenic analysis are shown in the Supplementary Files.

### 4.3. Conservation and Structural Analysis

Protein sequences were acquired from the UniProt website online (https://www.uniprot.org, accessed on 10 August 2022). Conservation analysis was conducted with the T-COFFEE Multiple Sequence Alignment Server (https://tcoffee.crg.eu, accessed on 15 August 2022). Protein secondary structure prediction was completed using PsiPred 3.3 (http://bioinf.cs.ucl.ac.uk/psipred, accessed on 17 August 2022). The three-dimensional structure was analyzed with the SWISS-MODEL website (https://swissmodel.expasy.org, accessed on 17 August 2022), and figures were prepared using the PyMOL Molecular Graphics System version 2.4 ( Schrodinger, New York, NY, USA).

### 4.4. Construction of Plasmids

The full-length cDNA sequences of wild-type and mutant (c.G143A) TMEM119 were cloned into pcDNA3.1, vector tagged with FLAG-tag and transfected into MC3T3-E1 cells, named as pcDNA3.1-TMEM119-FLAG-WT (WT) and pcDNA3.1-TMEM119-FLAG-MUT (MUT). Empty vector (pcDNA3.1-FLAG, EV) was used as the control. The pcDNA3.1-ATF4 plasmid was constructed for immunofluorescence, co-immunoprecipitation and rescue assays. The entire coding sequences of the plasmids were confirmed by DNA sequencing.

### 4.5. Cell Culture and Liposome Transfection

The mouse pre-osteoblastic MC3T3-E1 cell line was cultured in α-MEM (Hyclone, Logan, UT, USA) supplemented with 10% fetal bovine serum (Analysis Quiz, Beijing, China) and 1% penicillin-streptomycin (Hyclone, Logan, UT, USA). Cells were cultured at 37 °C in a humidified atmosphere with 5% CO_2_ and 95% air in an incubator. Every two days, the culture medium was changed.

After exceeding 70% cell confluence, transient plasmid transfection was conducted using Lipofectamine 8000 (Beyotime, Shanghai, China) based on the manufacturer’s protocol. Western blot assay and qPCR were used to evaluate transfection efficiency and gene expression.

### 4.6. Quantitative Real-Time Polymerase Chain Reaction (qRT-PCR)

Cellular total RNA was extracted using TRIzol reagent (Invitrogen, Carlsbad, CA, USA). cDNA was synthesized using Hiscript III RT SuperMix for the qPCR Kit (Vazyme, Nanjing, China). We used DNase to digest RNA in order to avoid genomic DNA contamination before we performed cDNA synthesis reaction. After 7 days of osteogenic induction in vitro, specific genes were detected by qPCR using an ABI Prism 7000 sequence detection system (Thermo Fisher Scientific Inc. Waltham, MA, USA) according to the manufacturer’s instructions of Taq Pro Universal SYBR qPCR Master Mix (Vazyme, Nanjing, China). Related mRNA expression, transmembrane protein 119 (*Tmem119*), osterix (*Osx*), bone sialoprotein 2 (*Bsp2*), osteocalcin (*Ocn*), runt-related transcription factor 2 (*Runx2*), glucose transporter type 1 (*Glut1*), hexokinase 1 (*Hk1*), lactate dehydrogenase A (*Ldha*), pyruvate kinase M2 (*Pkm2*), glucose-6-phosphate dehydrogenase (*G6pd*), activating transcription factor 4 (*Atf4*) and phosphoglycerate kinase 1 (*Pgk1*) were calculated using the 2-ΔΔCt method. GADPH and β-actin served as reference controls. The primer sequences are listed in Supplementary Table S7. Three replicates were carried out for each run.

### 4.7. Western Blot Analysis

At the indicated time after transfection, the cells were collected and lysed in RIPA buffer (Solarbio, Beijing, China) containing 100 μM phenylmethylsulfonyl fluoride (PMSF, Solarbio, Beijing, China) on ice. Based on the manufacturer’s protocol, protein from the cytoplasm and nuclei was separated with a Nuclear and Cytoplasmic Protein Extraction Kit (Beyotime, Shanghai, China). Protein concentration was determined with the BCA Protein Assay Kit (Thermo Fisher, Waltham, MA, USA). Following SDS-PAGE electrophoretic separation, proteins were transferred to polyvinylidene difluoride (PVDF) membranes. The protein-bound membrane was blocked in 5% skimmed milk (Solarbio, Beijing, China) for 1 h at room temperature and then incubated with transmembrane protein 119 (TMEM119, 1:1000, 66948-1-Ig; Proteintech, Wuhan, China), osterix (OSX, 1:1000, A18699; Abclonal, Wuhan, China), one sialoprotein 2 (BSP2, 1:1000, A16220; Abclonal, Wuhan, China), runt-related transcription factor 2 (RUNX2, 1:1000, A2851; Abclonal, Wuhan, China), FLAG-tag (DYKDDDDK-Tag, 1:1000, MA1-91878; Invitrogen, Carlsbad, CA, USA), activating transcription factor 4 (ATF4, 1:1000, A0201; Abclonal, Wuhan, China), glucose transporter type 1 (GLUT1, 1:1000, 380464; ZEN-BIOSCIENCE, Chengdu, China), hexokinase 1 (HK1, 1:1000, 222320; ZEN-BIOSCIENCE, Chengdu, China), pyruvate kinase M2 (PKM2, 1:1000, 321004; ZEN-BIOSCIENCE, Chengdu, China), lactate dehydrogenase A (LDHA, 1:1000, 380954; ZEN-BIOSCIENCE, Chengdu, China), GAPDH (1:1000, AF0006; Beyotime, Shanghai, China) and β-actin (1:1000, AF0003; Beyotime, Shanghai, China) antibodies overnight at 4 °C. The membranes were incubated with the appropriate horseradish peroxidase (HRP)-conjugated secondary antibodies (1:1000, A0216 and A0208; Beyotime, Shanghai, China) for 1 h at room temperature, and then the antigen-antibody complexes were visualized using enhanced chemiluminescent (ECL) solution (NCM Biotech, Suzhou, China).

### 4.8. In Vitro Mineralization Ability Detection

For mineralized induction, osteoblast differentiation medium (OM) was used, supplemented with 10 nM dexamethasone, 10 mM β-glycerophosphate and 50 µg/mL of L-ascorbic acid. The mineralization ability of MC3T3-E1 cells was quantified using qRT-PCR, Western blot, alkaline phosphatase staining and alizarin red staining. After confluent cells were grown in OM medium for 1 week, the cells were fixed with 4% fixative solution (Solarbio, Beijing, China) for 30 min and stained with the BCIP/NBT Alkaline Phosphatase Color Development Kit (Beyotime, Shanghai, China) and alizarin red (Solarbio, Beijing, China) according to the manufacturer’s instructions to detect mineralization.

### 4.9. Cell Immunofluorescence

The 4 × 10^4^ cells were seeded on a sterile glass coverslip in each well of 24-well plates with 1 mL medium. At 48 h after transfection, coverslip-grown MC3T3-E1 cells were rinsed with PBS for three times and then fixed in 4% fixative solution for 30 min. Then, the coverslips were incubated with TMEM119 and ATF4 antibody overnight at 4 °C. Following incubation of the secondary FITC-labeled goat anti-mice IgG (H+L) (Beyotime, Shanghai, China) or RBITC-labeled goat anti-rabbit IgG (Solarbio, Beijing, China) antibodies and DAPI (Sigma, St Louis, MO, USA), the coverslips were installed on glass slides. Images were obtained using a TCS-SP8 STED 3X confocal imaging system (Leica, Wetzlar, Germany) and APO 63×/1.4 oil objective.

### 4.10. Co-Immunoprecipitation (Co-IP)

The MC3T3-E1 cells were grown in 10 cm dishes for 48 h after plasmid transfection. Cells were collected and lysed as mentioned above. According to the manufacturer’s instructions, aliquot of the supernatant containing 1 mg of protein was clarified and incubated with 40 μL protein A + G-agarose beads (Beyotime, Shanghai, China) on a rocking platform at 4 °C overnight. The beads were collected and washed three times with PBS, resuspended in 40 μL 2× loading buffer and boiled for 10 min. Immunoprecipitated proteins were subjected to Western blot analysis using anti-TMEM119 and anti-ATF4 antibodies.

### 4.11. Cell Proliferation Assay

MC3T3-E1 cells were seeded at 3 × 10^3^/well in 96-well plates with 100 μL medium. The next day, transfection was performed, and cell proliferative activity was assessed using a Cell Counting Kit-8 (CCK-8; NCM Biotech, Suzhou, China) at 1, 3, 5 and 7 days after transfection according to the standard protocol. Assay solution was prepared at a ratio of 10 μL CCK-8 regent per 100 μL medium, then 100 μL assay solution was added per well, and the solution was incubated for 1 h before testing. Absorbance levels at 450 nm were measured by a microplate reader (ELX808, BioTek, Winooski, VT, USA).

### 4.12. Wound-Healing Assay

The center of the bottom of the 6-well plate was marked with marker. The 2 × 10^5^ cells were inoculated in each well of 6-well plates with 2 mL growth medium and cultured in an incubator until the cell monolayer was confluent. The next day, a scratch was performed with 200 μL pipette tips perpendicular to the mark to establish an in vitro cell wound model. After rinsing the detached cell clusters with PBS three times, pictures of the same area were recorded under a microscope at 0 and 12 h after the scratch. ImageJ software version 2.0.0 (NIH, Bethesda, MD, USA) was used to calculate the scratch area, and the scratch-healing rate = (0 h scratch area − indicated time point scratch area)/0 h scratch area × 100%.

### 4.13. Small Interfering RNA (siRNA) Construction and Transfection

Mice ATF4 siRNA (si-ATF4, sense, GGAGUUUAAGCAGGAGCAUTT, antisense, AUGCUCCUGCUUAAACUCCTT) and control siRNA (siNC) were constructed by RiboBio Co., Ltd. and were transfected by the procedures recommended using Lipo8000. The knockdown effect was verified by qPCR and Western blot. Cells were osteogenically induced for 7 days after co-transfection of siATF4 and TMEM119 plasmids. Cell mineralization function was detected by Western blot, ALP and alizarin red staining assays. Cell glycolytic function was detected by lactic acid and glucose measurement, qPCR, Western blot and Seahorse energy assays.

### 4.14. Glycolytic Rate Assay and Mitochondrial Stress Test

The 4 × 10^4^ MC3T3-E1 cells were plated per well in 24-well Seahorse V7 culture plates (Agilent Seahorse catalog # 100777-004; Agilent, Santa Clara, CA, USA) with 250 μL of growth medium. One day after plating, transfection was performed, and growth medium was replaced with differentiation medium. After osteogenic differentiation for 7 days, we measured the extracellular acidification rate (ECAR) and oxygen consumption rate (OCR) on a Seahorse XF24 (Agilent, Santa Clara, CA, USA), as described by the manufacturer.

### 4.15. Measurement of Glucose in Culture Medium

Cells were transfected and osteo-inducted for 7 days in 6-well plates, and then 1 mL culture medium was collected from each group to detect glucose content. According to the protocol of the Glucose Assay Kit (Njjcbio, Nanjing, China), 250 μL working solution and 2.5 μL sample medium were added in each well of a 96-well plate and placed in an incubator at 37 °C for 10 min. Optical density (OD) was measured at a wavelength of 505 nm (OD505 value) using a microplate reader. Glucose content (mmol/L) = (measured OD505 value − blank control OD505 value)/(standard OD505 value − blank control OD505 value) × glucose concentration of standard solution × sample dilution ratio. Each treatment group had 3 parallel wells.

### 4.16. Measurement of Lactic Acid in Culture Medium

Cells were transfected and osteo-inducted for 7 days in 6-well plates, and then 1 mL culture medium was collected from each group to detect glucose content. According to the protocol of the Glucose Assay Kit (Njjcbio, Nanjing, China), 250 μL of working solution and 2.5 μL of sample medium were added in each well of a 96-well plate and placed in incubator at 37 °C for 10 min. Optical density (OD) was measured at a wavelength of 505 nm (OD505 value) using a microplate reader. Glucose content (mmol/L) = (measured OD505 value − blank control OD505 value)/(standard OD505 value − blank control OD505 value) × glucose concentration of standard solution × sample dilution ratio. Each treatment group had 3 parallel wells.

### 4.17. Mouse Treatment and Tissue Preparation

The murine experiment was conducted on pregnant female ICR mice (6–8 weeks old) and postnatal ICR mice (sex randomization). All mice were obtained from Beijing Vitalstar Biotechnology Co., Ltd. (Beijing, China), and each mouse received standard feed and water in a separate cage with a 12 h light/dark cycle and a constant room temperature. Animal experiments were performed in the animal laboratory of Peking University’s School of Stomatology. All animal experiments were approved by the Peking University Biomedical Ethics Committee (approval number: LA2021165) and performed according to the guidelines of the Peking University Animal Ethics Committee. The mice were caged in a 2:1 male/female ratio. The middle of the day following the detection of a vaginal plug was designated as embryonic day 0.5 (E0.5), and the middle of the day of birth was taken as postnatal day 0.5 (P0.5). The animals were divided into 8 groups, including fetal mice at different embryonic days (E13.5, E14.5, E15.5, E17.5, 9 embryos from 3 different pregnant mice) and postnatal mice at different stages (P1.5, P5.5, P9.5, P13.5, n = 3 for each group). In this study, we did not perform any extra treatment on the mice and observed the development and eruption of molar germs and the expression of the target protein under physiological conditions, then compared the protein expression level between groups. The vital signs of mice were stable before sampling, and then mice were sacrificed by cervical dislocation under 2% pentobarbital sodium anesthesia on specific days. Embryos were dissected from the uterus into cold PBS (pH 7.4). The heads from these embryos and mandibles from postnatal mice were dissected under stereomicroscope (Leica, Wetzlar, Germany) and fixed in 4% paraformaldehyde in 0.1 M phosphate buffer (pH 7.4) at 4 °C for 24 h, followed by embedding in paraffin. The head diameters of fetal mice were measured after separation, and the error of each sample in one group was not more than 1 mm. Mandibular samples of postnatal mice were decalcified with 10% EDTA (pH7.2) for 2–14 days until complete decalcification. Tissues were serially sectioned at a thickness of 5 μm in the sagittal (mandible samples) or coronal (head samples) plane.

### 4.18. Tissue Immunohistochemistry and Immunofluorescence

After deparaffinization and rehydration, sections were boiled in a microwave for 20 min in Tris–EDTA buffer (pH 9.5) (ZSGB-BIO, Beijing, China) for antigen retrieval. Slices were treated with 3% hydrogen peroxide (H_2_O_2_) solution for 20 min at room temperature to block the endogenous peroxidase activity and subsequently incubated with normal goat serum (ZSGB-BIO, Beijing, China) for 30 min at room temperature. Tissue sections were incubated with the rabbit anti-TMEM119 monoclonal antibody (1:250, ab209064; Abcam, Cambridge, UK) overnight at 4 °C.

In tissue immunohistochemistry, slices were incubated with HRP-labeled goat anti-rabbit IgG (H+L) (1:50, A0208; Beyotime, Shanghai, China) for 1 h at room temperature. Immunostained positive cells were then visualized using diaminobenzidine tetrahydrochloride solution (DAB, ALI-9017, ASGB-BIO, Beijing, China). Finally, the sections were counterstained with hematoxylin and mounted. The sections were washed three times for 5 min in PBS following each incubation step. The brain tissues in the section served as positive controls for the TMEM119 antibody. Negative control tests were carried out using PBS instead of primary antibodies to establish the specificity of the immunostaining.

In tissue immunofluorescence, the slices were incubated with RBITC-labeled goat anti-rabbit IgG (Solarbio, Beijing, China) for 1 h at room temperature and mounted using mounting medium (with DAPI) (Solarbio, Beijing, China). The sections were washed three times for 5 min in PBS following each incubation step. Images were obtained using a TCS-SP8 STED 3× (Leica, Wetzlar, Germany) confocal imaging system and APO 20× oil objective.

### 4.19. Statistical Analysis

Three replicates of each experiment were conducted. Data were collected, stored and managed in a spreadsheet using Microsoft Excel 2010 software. Data were analyzed, and figures prepared, using SPSS version 21.0 (IBM Inc., Armonk, NY, USA) and GraphPad Prism software version 5.04 (San Diego, CA, USA). Data were expressed as the number and percentage of subjects (N (%)) or mean ± standard deviation (SD) and illustrated using bar charts showing the standard deviations. Therefore, one-way ANOVA and independent *t*-test were performed to test the differences between the groups for normally distributed data. Additionally, Pearson’s correlation coefficient was used for normally distributed data. A *p* value < 0.05 was taken to represent a significant difference.

## 5. Conclusions

The TMEM119 (c.G143A, p.S48L) mutation is a potential pathogenic gene of the PFE family, operating through affecting the glucose metabolism and mineralization function of osteoblasts under the regulation of ATF4.

## Figures and Tables

**Table 1:** Clinical and molecular features of primary failure of eruption (PFE) individuals.

Patient	Sex (F/M)	Clinical Feature of PFE	Mutation			
			Nucleotide	Codon	Type	Location
I-2,II-2	F	Doubtful	Unknown	Unknown	Unknown	Unknown
II-3	M	Doubtful	Unknown	Unknown	Unknown	Unknown
III-1	F	Yes	143G > A	S48L	Missense	Extra-membrane
IV-1	F	Yes	143G > A	S48L	Missense	Extra-membrane
Control 1	F	No	Wild-type	Normal	Normal	Extra-membrane
Control 2	M	No	Wild-type	Normal	Normal	Extra-membrane

F, Female; M, Male.

## Data Availability

All datasets generated for this study are included in the article/Supplementary Materials.

## Supplementary Materials

The following supporting information can be downloaded at: https://www.mdpi.com/article/10.3390/ijms25052821/s1.
