# Supplementary material for: TMEM119 (c.G143A, p.S48L) Mutation Is Involved in Primary Failure of Eruption by Attenuating Glycolysis-Mediated Osteogenesis
Source: Int J Mol Sci. 2024 Feb 29;25(5):2821. doi: 10.3390/ijms25052821 (PMC10931804; doi:10.3390/ijms25052821)
Supplement: Supplementary file 1 [file ijms-25-02821-s001.zip › ijms-2866861-supplementary.pdf]

SUPPLEMENTARY MATERIAL

Mutation Screening

The common genetic mutation of two patients from the family were detected by whole-exome sequencing (WES) conducted by Tsingke Biotechnology Co., Ltd. (Beijing, China), which refers to a genome analysis method that uses sequence capture technology to concentrate DNA in all exon regions of the genome for high-throughput sequencing analysis. Quality control, alignment analysis, and the calling analysis of WES data was shown in Supplementary Table S3 and Supplementary Table S4. The possible mutation must be variants with a minor allele frequency (MAF) <1% as reported in available databases (1000 genomes and gnomAD) and alter the original amino acid sequence such as missense mutation, deletion or insertion. At the same time, the mutation should be shared by both patients but not by the healthy controls. Based on these conditions, we obtained 4 SNPs, including transmembrane protein 119 (TMEM119), family with sequence similarity 83 member G (FAM83G), desmoplakin (DSP), sperm associated antigen 17 (SPAG17), and 2 Indel mutant genes, including proprotein convertase subtilisin/kexin type 4 (PCSK4), transmembrane protein 39A (TMEM39A). Subsequently, according to gene function, we screened out three candidate genes, including TMEM119, PCSK4 and FAM83G, may related to osteogenesis, osteoclast, calcium and phosphorus metabolism and tooth development. To verify the accuracy of whole-exon sequencing results, we utilized Sanger sequencing. 600 bp DNA sequence around mutant site was amplified in both patients and healthy controls with polymerase chain reaction (PCR). The primer sequences were as follows: TMEM119, forward,5'-ATCACGTACTGGCGGAAGAA-3', and reverse, 5'- GAGTTCCGCCATGACTTCTCT-3' (product size 431bp); PCSK4, forward,5'- CTACACGCTGCTGCTCTATG-3', and reverse, 5'-AGCCTTGTGTGGTTGAAGAA-3' (product size 334bp); FAM83G， forward, 5'- GAGCAGGCAGGTACTCGAAG-3', and reverse, 5'- AGCTGGGACTGCAACTAGAAC-3' (product size 456bp). PCR products were evaluated by agarose gel electrophoresis shown as Supplementary Figure S3 and sequenced in both forward and reverse directions by Tsingke Biotechnology Co. Ltd. (Beijing, China). Finally, the priority of candidate genes was sequenced using ToppGene

(https://toppgene.cchmc.org/prioritization.jsp). Training genes, test genes and test results were shown in Supplementary Table S5 and Supplementary Table S6. Based on the above analysis, TMEM119 was selected as the candidate disease gene for the family.

Pathogenic analysis

We used the T-COFFEE Multiple Sequence Alignment Server (https://tcoffee.crg.eu/) to align the orthologous sequences of TMEM119 downloaded from UniProt (http://www.uniprot.org): Homo sapiens, Mus musculus, Rattus norvegcus, Gorrilla, Pan troglodytes, and Oryctolagus cuniculus. The PSIPRED online server (http://biinf.cs.ucl.ac.uk/psipred) and SWISS-MODEL online server （https://swissmodel.expasy.org） were applied to construct the secondary and 3D structures of the wild-type and Ser48Leu mutant TMEM119. Predicted structures referred to the structure displayed in PyMol software 2.5 (Schrodinger, New York, NY, USA). The potential impact on the structure and function of the corresponding protein was predicted by SIFT（http://sift.bii.a-star.edu.sg/）, Polyphen（PolyPhen-2, http://genetics.bwh.harvard.edu/pph2/), Mutation Assessor（http://mutationassessor.org/）and Mutation Taster（http: //www.mutationtaster.org/）as shown in Supplementary Table S1.

Supplementary Table S1 The results of variation prediction software

Prediction software	Prediction score	Reference score
SIFT	0.02	Damaging
PolyPhen-2	0.975	Probably damaging
Mutation Assessor	2.075	Moderately affected function
Mutation Taster	0.988	Damaging

Supplementary Table S2 All mutations located in PTH, PTH1R and PTHrP genes in the family

Start	End	Ref	Alt	Func.refGene	Gene.refGene		1000g2015aug_all	1000g2015aug_eas	Exoni-cFunc.refGene	Control1	Control2	II-1	III-1
46907435	46907435	A	G	intergenic	MYL3,PTH1R	rs3762795	0.469848	0.381		0/1:2,2:4:37:37,0,37	1/1:0,1:1:3:43,3,0	1/1:0,1:1:3:43,3,0	/
46933266	46933266	G	T	intronic	PTH1R	rs1466275	0.449681	0.4365		1/1:0,4:4:12:111,12,0	1/1:0,1:1:3:41,3,0	1/1:0,1:1:3:41,3,0	/
46934292	46934292	C	A	intronic	PTH1R	rs4683300	0.458466	0.4395		0/1:5,1:6:23:23,0,138	0/1:1,7:8:12:251,0,12	0/1:10,3:13:86:86,0,312	1/1:0,1:1:3:42,3,0
46935681	46935681	C	T	intronic	PTH1R	rs724449	0.458067	0.4395		0/1:4,3:7:96:96,0,126	0/1:4,1:5:25:25,0,133	0/1:2,2:4:50:50,0,65	1/1:0,5:5:15:162,15,0
46941116	46941116	A	G	intronic	PTH1R	rs2242116	0.502796	0.4425		1/1:0,2:2:6:72,6,0	0/1:3,2:5:67:67,0,103	0/0:2,0:2:6:0,6,49	1/1:0,1:1:3:43,3,0
46941626	46941626	T	C	intronic	PTH1R	rs10049237	0.789936	0.6091		/	1/1:0,2:2:6:76,6,0	/	/
46944274	46944274	T	C	exonic	PTH1R	rs1138518	0.643371	0.4425	synonymous SNV	0/1:67,62:129:99:1744,0,1954	0/1:87,67:154:99:1844,0,2317	0/1:100,74:174:99:2032,0,2672	1/1:0,169:169:99:5385,507,0
46948526	46948526	T	C	intergenic	PTH1R,CCDC12	rs7652849	0.966054	0.998		/	1/1:0,7:7:21:234,21,0	1/1:0,6:6:18:148,18,0	1/1:0,1:1:3:43,3,0
46951935	46951935	T	C	intergenic	PTH1R,CCDC12	rs11719795	0.664337	0.4385		/	/	1/1:0,2:2:6:49,6,0	1/1:0,1:1:3:41,3,0

46957298	46957298	G	A	intergenic	PTH1R,CCDC12	rs9855938	0.563299	0.4375	/	/	/	1/1:0,3:3:9:87,9,0
46937683	46937686	AGAC	-	intronic	PTH1R	rs138467732	0.458067	0.4385	0/0:3,0:3:9:0,9,182	/	0/1:1,2:3:52:81,0,52	1/1:0,4:4:12:180,12,0
46945263	46945263	-	A	UTR3	PTH1R	rs142021243	0.153155	0.1825	0/0:19,1:20:32:0,32,406	0/1:12,12:24:99:246,0,214	0/1:19,20:39:99:413,0,368	0/1:9,8:17:99:151,0,159
46949036	46949039	TGTA	-	intergenic	PTH1R,CCDC12	rs10579601	0.454273	0.4385	1/1:0,2:2:6:90,6,0	0/0:1,0:1:3:0,3,45	/	/
46949123	46949126	TGTG	-	intergenic	PTH1R,CCDC12	rs10587792	0.621406	0.4663	1/1:0,3:3:9:135,9,0	/	/	/
46955971	46955971	A	-	intergenic	PTH1R,CCDC12	rs397989442	0.652955	0.4385	1/1:0,4:4:12:99,12,0	0/0:1,0:1:3:0,3,27	/	1/1:0,2:2:6:51,6,0
13492898	13492898	A	G	intergenic	BTBD10,PTH	rs307234	0.915535	0.999	1/1:0,2:2:6:84,6,0	/	/	/
13492952	13492952	A	G	intergenic	BTBD10,PTH	rs307235	0.986621	1	1/1:0,3:3:9:90,9,0	/	/	/
13493272	13493272	T	C	intergenic	BTBD10,PTH	rs307236	0.296526	0.5208	1/1:0,3:3:9:89,9,0	/	/	0/0:1,0:1:3:0,3,10
13497153	13497153	C	T	intergenic	BTBD10,PTH	rs12365769	0.151558	0.1706	/	/	1/1:0,2:2:6:79,6,0	0/0:2,0:2:6:0,6,49
13497179	13497179	T	C	intergenic	BTBD10,PTH	rs12361526	0.151558	0.1706	/	/	1/1:0,2:2:6:82,6,0	0/0:2,0:2:6:0,6,49
13500276	13500276	T	C	intergenic	BTBD10,PTH	rs307215	0.914337	0.999	/	1/1:0,2:2:6:84,6,0	/	/

13501825	13501825	A	G	intergenic	BTBD10,PTH	rs307214	0.986222	1		1/1:0,7:7:21:211,21,0	1/1:0,2:2:6:75,6,0	1/1:0,2:2:6:49,6,0	/
13502412	13502412	A	C	intergenic	BTBD10,PTH	rs307213	0.914337	0.999		/	1/1:0,2:2:6:84,6,0	/	/
13508240	13508240	G	C	intergenic	BTBD10,PTH	rs2004314	0.456869	0.3085		0/0:1,0:1:3:0,3,42	0/0:6,0:6:18:0,18,133	0/0:2,0:2:6:0,6,49	0/1:4,5:9:90:115,0,90
13508320	13508320	T	C	intergenic	BTBD10,PTH	rs307255	0.914137	0.999		/	1/1:0,4:4:12:112,12,0	1/1:0,6:6:18:184,18,0	1/1:0,7:7:21:226,21,0
13510474	13510474	G	A	intergenic	BTBD10,PTH	rs307252	0.507188	0.6935		/	1/1:0,2:2:6:78,6,0	/	0/0:1,0:1:3:0,3,38
13511359	13511359	G	A	intergenic	BTBD10,PTH	rs307251	0.279553	0.5228		/	1/1:0,2:2:6:78,6,0	/	0/0:1,0:1:3:0,3,41
13514053	13514053	G	T	exonic	PTH	rs6256	0.151358	0.121	synonymous SNV	0/0:55,0:55:99:0,164,1821	0/0:77,0:77:99:0,231,2557	0/0:104,0:104:99:0,312,3409	0/1:32,34:66:99:926,0,779
13514417	13514417	C	T	intronic	PTH	rs694	0.417732	0.3065		0/1:6,6:12:99:190,0,175	0/0:15,0:15:45:0,45,612	0/1:8,7:15:99:183,0,258	0/1:4,6:10:99:199,0,135
13514505	13514505	C	T	intronic	PTH	rs177706	0.497005	0.6935		0/1:1,1:2:31:34,0,31	1/1:0,6:6:18:223,18,0	0/1:1,3:4:26:107,0,26	0/0:2,0:2:6:0,6,76
13521469	13521469	C	T	intergenic	PTH,FAR1	rs2121633	0.417732	0.3046		1/1:0,1:1:3:39,3,0	/	1/1:0,2:2:6:49,6,0	/
13522997	13522997	G	A	intergenic	PTH,FAR1	rs307209	0.986422	1		1/1:0,1:1:3:37,3,0	/	1/1:0,5:5:15:163,15,0	/

13539512	13539512	T	C	intergenic	PTH,FAR1	rs1459017	0.915335	0.999	/	1/1:0,2:2:6:90,6,0	/	/
13548662	13548662	A	G	intergenic	PTH,FAR1	rs11022868	0.43131	0.3046	1/1:0,3:3:9:121,9,0	0/0:5,0:5:15:0,15,135	/	0/1:1,1:2:35:35,0,35
13555520	13555520	A	G	intergenic	PTH,FAR1	rs1841151	0.993211	1	1/1:0,3:3:9:124,9,0	1/1:0,4:4:12:99,12,0	1/1:0,5:5:15:142,15,0	1/1:0,1:1:3:43,3,0
13557255	13557255	T	C	intergenic	PTH,FAR1	rs1459014	0.153355	0.1706	/	/	0/1:1,1:2:19:19,0,19	1/1:0,3:3:9:74,9,0
13558262	13558262	C	T	intergenic	PTH,FAR1	rs11022870	0.451877	0.6944	/	/	/	0/1:1,2:3:31:71,0,31
13568618	13568618	A	C	intergenic	PTH,FAR1	rs11022874	0.452077	0.6954	/	1/1:0,4:4:12:99,12,0	/	0/1:1,1:2:32:32,0,36
13571050	13571050	G	A	intergenic	PTH,FAR1	rs4757165	0.57488	0.6954	/	1/1:0,2:2:6:78,6,0	1/1:0,2:2:6:49,6,0	/
13571113	13571113	A	G	intergenic	PTH,FAR1	rs4757166	0.451677	0.6944	/	1/1:0,1:1:3:41,3,0	1/1:0,2:2:6:49,6,0	/
13571211	13571211	T	G	intergenic	PTH,FAR1	rs4757167	0.451877	0.6944	/	1/1:0,4:4:12:149,12,0	1/1:0,2:2:6:49,6,0	/
13574774	13574774	A	G	intergenic	PTH,FAR1	rs4757170	0.154752	0.1696	0/0:1,0:1:3:0,3,25	0/0:1,0:1:3:0,3,38	0/1:1,2:3:1:78,0,1	0/0:1,0:1:3:0,3,32
13574915	13574915	G	A	intergenic	PTH,FAR1	rs4757171	0.574481	0.6954	1/1:0,1:1:3:42,3,0	1/1:0,3:3:9:116,9,0	1/1:0,2:2:6:81,6,0	/
13582329	13582329	G	A	intergenic	PTH,FAR1	rs2085652	0.452077	0.6944	/	1/1:0,2:2:6:49,6,0	/	0/1:1,4:5:23:84,0,23

13582441	13582441	A	G	intergenic	PTH,FAR1	rs7107041	0.915136	0.999	/	1/1:0,1:1:3:42,3,0	/	1/1:0,2:2:6:87,6,0
13588150	13588150	C	A	intergenic	PTH,FAR1	rs4757179	0.986022	0.999	1/1:0,1:1:3:40,3,0	1/1:0,2:2:6:49,6,0	1/1:0,2:2:6:49,6,0	/
13601682	13601682	C	T	intergenic	PTH,FAR1	rs4757184	0.452077	0.6944	1/1:0,2:2:6:49,6,0	/	/	1/1:0,1:1:3:40,3,0
13605157	13605157	C	A	intergenic	PTH,FAR1	rs183597485	0.003794	0.0188	0/1:2,3:5:34:65,0,34	0/0:3,0:3:9:0,9,120	1/1:0,4:4:12:129,12,0	/
13605232	13605232	C	A	intergenic	PTH,FAR1	rs11493157	0.452476	0.6944	0/1:2,2:4:65:65,0,70	1/1:0,6:6:18:190,18,0	0/0:5,0:5:15:0,15,142	/
13612518	13612518	C	T	intergenic	PTH,FAR1	rs7129881	0.452676	0.6944	0/1:4,1:5:25:25,0,147	1/1:0,4:4:12:150,12,0	/	0/1:5,2:7:28:28,0,120
13612552	13612552	A	G	intergenic	PTH,FAR1	rs7115286	0.872404	0.999	1/1:0,6:6:18:176,18,0	1/1:0,5:5:15:159,15,0	/	1/1:0,8:8:24:198,24,0
13612585	13612585	C	A	intergenic	PTH,FAR1	rs7129905	0.444888	0.6944	0/0:4,0:4:12:0,12,128	1/1:0,4:4:12:150,12,0	/	0/1:3,1:4:26:26,0,62
13613489	13613489	A	G	intergenic	PTH,FAR1	rs7941196	0.452276	0.6944	0/0:1,0:1:3:0,3,31	1/1:0,2:2:6:83,6,0	1/1:0,1:1:3:44,3,0	/
13617366	13617366	G	T	intergenic	PTH,FAR1	rs10766102	0.531949	0.5595	1/1:0,1:1:3:38,3,0	/	0/0:1,0:1:3:0,3,43	1/1:0,2:2:6:49,6,0
13619440	13619440	G	C	intergenic	PTH,FAR1	rs28613793	0.979433	1	1/1:0,2:2:6:49,6,0	1/1:0,1:1:3:39,3,0	1/1:0,1:1:3:43,3,0	/
13620172	13620172	T	C	intergenic	PTH,FAR1	rs9630182	0.539137	0.5645	0/1:1,5:6:20:118,0,20	0/0:1,0:1:3:0,3,39	/	1/1:0,1:1:3:43,3,0

13620302	13620302	A	G	intergenic	PTH,FAR1	rs1502239	0.539137	0.5645	1/1:0,2:2:6:82,6,0	0/0:1,0:1:3:0,3,41	/	/
13623179	13623179	C	T	intergenic	PTH,FAR1	rs10832079	0.277955	0.2609	1/1:0,3:3:9:88,9,0	0/0:1,0:1:3:0,3,39	/	0/0:1,0:1:3:0,3,41
13624421	13624421	C	G	intergenic	PTH,FAR1	rs10832080	0.209665	0.2599	1/1:0,3:3:9:90,9,0	/	/	/
13679433	13679433	T	C	intergenic	PTH,FAR1	rs2699445	0.977636	1	/	1/1:0,3:3:97,9,0	1/1:0,1:1:3:38,3,0	/
13493204	13493204	-	A	intergenic	BTBD10,PTH	rs397731498	0.436502	0.2946	1/1:0,2:2:6:50,6,0	/	/	1/1:0,1:1:3:25,3,0
13502272	13502272	C	-	intergenic	BTBD10,PTH	/	.	.	/	0/0:2,0:2:6:0,6,74	/	1/1:0,2:2:6:75,6,0
13558331	13558333	CTC	-	intergenic	PTH,FAR1	rs35940563	0.451877	0.6944	/	/	0/0:3,0:3:9:0,9,135	0/1:2,3:5:85:120,0,85
13565023	13565023	T	-	intergenic	PTH,FAR1	/	.	.	1/1:0,3:3:9:70,9,0	/	0/0:1,0:1:3:0,3,10	/
13589642	13589642	-	G	intergenic	PTH,FAR1	rs397848567	0.453674	0.6944	0/0:2,0:2:6:0,6,74	1/1:0,3:3:9:111,9,0	1/1:0,1:1:3:37,3,0	0/0:2,0:2:6:0,6,74
13650878	13650878	T	-	intergenic	PTH,FAR1	rs34458047	0.746605	0.9435	0/0:2,0:2:6:0,6,55	1/1:0,2:2:6:50,6,0	1/1:0,1:1:3:24,3,0	/
13683389	13683390	TT	-	intergenic	PTH,FAR1	/	/	/	1/1:0,2:2:6:63,6,0	0/0:1,0:1:3:0,3,20	0/1:1,2:3:25:55,0,25	/
27961587	27961587	T	C	intergenic	KLHL42,PTHRP	rs7312945	0.792931	0.7004	1/1:0,1:1:3:45,3,0	1/1:0,2:2:6:90,6,0	0/0:1,0:1:3:0,3,65	/

27961597	27961597	T	A	intergenic	KLHL42,PTHRP	rs7312948	0.792931	0.7004	1/1:0,1:1:3:45,3,0	1/1:0,2:2:6:90,6,0	0/0:1,0:1:3:0,3,65	/
27961793	27961793	T	G	intergenic	KLHL42,PTHRP	rs4931495	0.992612	1	1/1:0,2:2:6:86,6,0	1/1:0,2:2:6:90,6,0	1/1:0,3:3:9:115,9,0	/
27961803	27961803	C	T	intergenic	KLHL42,PTHRP	rs4931496	0.791334	0.6994	0/1:1,1:2:31:34,0,31	1/1:0,2:2:6:79,6,0	0/0:3,0:3:9:0,9,123	/
27963358	27963358	A	G	intergenic	KLHL42,PTHRP	rs4931500	0.895168	0.9325	1/1:0,4:4:12:161,12,0	1/1:0,2:2:6:84,6,0	1/1:0,1:1:3:44,3,0	1/1:0,5:5:15:174,15,0
27963402	27963402	G	A	intergenic	KLHL42,PTHRP	rs3751236	0.173922	0.3462	0/0:3,0:3:9:0,9,125	0/0:3,0:3:9:0,9,120	0/0:1,0:1:3:0,3,41	0/1:1,4:5:26:69,0,26
27963546	27963546	G	A	intergenic	KLHL42,PTHRP	rs2347223	0.509185	0.6458	0/0:4,0:4:12:0,12,164	1/1:0,2:2:6:77,6,0	/	/
27966210	27966210	C	G	intergenic	KLHL42,PTHRP	rs11610005	0.418331	0.4028	0/1:2,1:3:32:32,0,65	0/1:2,3:5:68:74,0,68	1/1:0,1:1:3:32,3,0	1/1:0,1:1:3:43,3,0
27969214	27969214	G	A	intergenic	KLHL42,PTHRP	rs11833076	0.727037	0.7738	1/1:0,4:4:12:136,12,0	1/1:0,2:2:6:49,6,0	/	/
27969591	27969591	G	C	intergenic	KLHL42,PTHRP	rs61915453	0.564097	0.5952	/	1/1:0,1:1:3:37,3,0	1/1:0,3:3:9:87,9,0	0/0:1,0:1:3:0,3,10
27983738	27983738	T	C	intergenic	KLHL42,PTHRP	rs2347229	0.833866	0.8323	1/1:0,3:3:9:59,9,0	1/1:0,1:1:3:41,3,0	/	/
27983816	27983816	C	A	intergenic	KLHL42,PTHRP	rs2347230	0.833866	0.8323	1/1:0,2:2:6:80,6,0	1/1:0,1:1:3:38,3,0	/	/
27985517	27985517	T	C	intergenic	KLHL42,PTHRP	rs5022769	0.6873	0.8185	1/1:0,2:2:6:82,6,0	/	0/0:2,0:2:6:0,6,49	0/0:1,0:1:3:0,3,43

27986072	27986072	C	T	intergenic	KLHL42,PTHRP	rs7297278	0.958267	1	1/1:0,4:4:12:127,12,0	1/1:0,5:5:15:143,15,0	1/1:0,1:1:3:33,3,0	/
27986108	27986108	A	T	intergenic	KLHL42,PTHRP	rs7297182	0.958466	1	1/1:0,5:5:15:225,15,0	1/1:0,4:4:12:180,12,0	1/1:0,1:1:3:45,3,0	1/1:0,2:2:6:90,6,0
27986113	27986113	A	G	intergenic	KLHL42,PTHRP	rs7297183	0.958466	1	1/1:0,5:5:15:225,15,0	1/1:0,4:4:12:180,12,0	1/1:0,1:1:3:45,3,0	1/1:0,2:2:6:90,6,0
27989399	27989399	C	T	intergenic	KLHL42,PTHRP	rs7310597	0.957668	1	1/1:0,1:1:3:39,3,0	1/1:0,4:4:12:129,12,0	1/1:0,1:1:3:40,3,0	1/1:0,1:1:3:34,3,0
27990590	27990590	C	T	intergenic	KLHL42,PTHRP	rs7315196	0.957668	1	/	/	1/1:0,2:2:6:80,6,0	1/1:0,3:3:9:89,9,0
27990653	27990653	T	C	intergenic	KLHL42,PTHRP	rs7300940	0.957668	1	/	1/1:0,3:3:9:135,9,0	1/1:0,2:2:6:90,6,0	1/1:0,1:1:3:45,3,0
27990665	27990665	G	A	intergenic	KLHL42,PTHRP	rs7315513	0.957668	1	/	1/1:0,3:3:9:135,9,0	1/1:0,2:2:6:90,6,0	1/1:0,1:1:3:45,3,0
27990692	27990692	G	A	intergenic	KLHL42,PTHRP	rs7315523	0.957668	1	/	1/1:0,3:3:9:89,9,0	1/1:0,4:4:12:139,12,0	1/1:0,1:1:3:38,3,0
27990726	27990726	T	C	intergenic	KLHL42,PTHRP	rs7301048	0.957268	1	/	1/1:0,4:4:12:99,12,0	1/1:0,2:2:6:78,6,0	/
28031526	28031526	C	T	intergenic	KLHL42,PTHRP	rs12301526	0.209265	0.1776	/	0/1:1,2:3:33:68,0,33	/	/
28034760	28034760	A	G	intergenic	KLHL42,PTHRP	rs258431	0.669129	0.7034	1/1:0,3:3:9:118,9,0	/	/	/
28049129	28049129	G	A	intergenic	KLHL42,PTHRP	rs258447	0.758986	0.5903	1/1:0,1:1:3:42,3,0	1/1:0,2:2:6:81,6,0	/	/

28049250	28049250	G	A	intergenic	KLHL42,PTHRP	rs258448	0.520567	0.4087	/	/	1/1:0,2:2:6:49,6,0	1/1:0,4:4:12:129,12,0
28051265	28051265	T	C	intergenic	KLHL42,PTHRP	rs258391	0.921725	0.8839	/	1/1:0,1:1:3:40,3,0	1/1:0,2:2:6:49,6,0	/
28063293	28063293	G	A	intergenic	KLHL42,PTHRP	rs12832168	0.90016	0.8839	/	1/1:0,2:2:6:77,6,0	1/1:0,1:1:3:40,3,0	1/1:0,2:2:6:49,6,0
28064019	28064019	A	G	intergenic	KLHL42,PTHRP	rs11049220	0.235024	0.1954	/	1/1:0,1:1:3:37,3,0	1/1:0,2:2:6:49,6,0	1/1:0,1:1:3:42,3,0
28064107	28064107	C	G	intergenic	KLHL42,PTHRP	rs11049221	0.406749	0.4742	/	1/1:0,5:5:15:124,15,0	1/1:0,3:3:9:93,9,0	1/1:0,2:2:6:84,6,0
28064235	28064235	T	C	intergenic	KLHL42,PTHRP	rs4931626	0.890775	0.8859	1/1:0,3:3:9:90,9,0	1/1:0,5:5:15:155,15,0	1/1:0,1:1:3:38,3,0	1/1:0,3:3:9:87,9,0
28066140	28066140	G	T	intergenic	KLHL42,PTHRP	rs3911839	0.909145	0.881	1/1:0,3:3:9:89,9,0	1/1:0,1:1:3:40,3,0	/	1/1:0,3:3:9:89,9,0
28071851	28071851	C	G	intergenic	KLHL42,PTHRP	rs7980330	0.89357	0.875	1/1:0,4:4:12:161,12,0	/	1/1:0,3:3:9:117,9,0	1/1:0,2:2:6:49,6,0
28071907	28071907	G	A	intergenic	KLHL42,PTHRP	rs7980518	0.419329	0.4306	1/1:0,5:5:15:165,15,0	0/0:2,0:2:6:0,6,71	1/1:0,5:5:15:170,15,0	0/0:6,0:6:18:0,18,172
28072126	28072126	T	C	intergenic	KLHL42,PTHRP	rs7970077	0.89377	0.875	/	1/1:0,1:1:3:39,3,0	1/1:0,3:3:9:89,9,0	1/1:0,1:1:3:40,3,0
28092594	28092594	C	T	intergenic	KLHL42,PTHRP	rs9651828	0.230431	0.1865	0/0:2,0:2:6:0,6,73	1/1:0,2:2:6:78,6,0	0/0:1,0:1:3:0,3,39	/
28092633	28092633	T	C	intergenic	KLHL42,PTHRP	rs9651829	0.230431	0.1855	0/0:2,0:2:6:0,6,76	1/1:0,2:2:6:64,6,0	0/0:1,0:1:3:0,3,39	/

28092791	28092791	G	A	intergenic	KLHL42,PTHRP	rs9651800	/	/		1/1:0,2:2:6:90,6,0	/	/	/
28093217	28093217	T	C	intergenic	KLHL42,PTHRP	rs2347590	0.238019	0.1845		/	1/1:0,2:2:6:84,6,0	/	1/1:0,1:1:3:43,3,0
28100899	28100899	A	G	intergenic	KLHL42,PTHRP	rs3894107	0.345048	0.3155		/	/	1/1:0,3:3:9:92,9,0	/
28104278	28104278	A	C	intergenic	KLHL42,PTHRP	rs10431271	0.307109	0.3155		0/0:1,0:1:3:0,3,40	/	0/1:2,4:6:31:114,0,31	1/1:0,2:2:6:49,6,0
28117127	28117127	C	G	intronic	PTHRP	rs40411	0.517772	0.5982		/	/	1/1:0,3:3:9:87,9,0	/
28117213	28117213	T	A	intronic	PTHRP	rs33087	0.437101	0.4415		/	/	1/1:0,3:3:9:135,9,0	/
28117497	28117497	T	A	intronic	PTHRP	rs33089	0.553315	0.5992		1/1:0,2:2:6:78,6,0	1/1:0,2:2:6:42,6,0	1/1:0,3:3:9:91,9,0	/
28122102	28122102	C	G	intronic	PTHRP	rs26302	0.662141	0.7302		1/1:0,2:2:6:84,6,0	1/1:0,5:5:15:190,15,0	1/1:0,2:2:6:80,6,0	/
28128075	28128075	C	T	intergenic	PTHRP,CCDC91	rs61915977	0.107628	0.1131		1/1:0,2:2:6:83,6,0	/	/	0/0:1,0:1:3:0,3,40
28128625	28128625	T	C	intergenic	PTHRP,CCDC91	rs12425376	0.469848	0.5784		0/1:2,7:9:51:186,0,51	0/0:10,0:10:29:0,29,303	0/1:3,1:4:29:29,0,77	1/1:0,1:1:3:41,3,0
28140253	28140253	T	G	intergenic	PTHRP,CCDC91	rs1314085	0.907149	0.8254		1/1:0,1:1:3:45,3,0	1/1:0,4:4:12:180,12,0	/	0/0:3,0:3:9:0,9,139
28140277	28140277	C	T	intergenic	PTHRP,CCDC91	rs1314084	0.750998	0.8254		1/1:0,1:1:3:45,3,0	1/1:0,4:4:12:180,12,0	/	0/0:2,0:2:6:0,6,99

28140675	28140675	G	A	intergenic	PTHRP,CCDC91	rs1267213	0.90595	0.8204	1/1:0,2:2:6:78,6,0	/	/	/
28140714	28140714	T	G	intergenic	PTHRP,CCDC91	rs1267212	0.90615	0.8214	1/1:0,4:4:12:120,12,0	1/1:0,1:1:3:38,3,0	/	0/0:1,0:1:3:0,3,38
28140829	28140829	C	T	intergenic	PTHRP,CCDC91	rs1267211	0.748802	0.8204	1/1:0,4:4:12:129,12,0	1/1:0,2:2:6:49,6,0	1/1:0,2:2:6:49,6,0	0/1:2,2:4:37:37,0,37
28140918	28140918	T	C	intergenic	PTHRP,CCDC91	rs1267210	0.90595	0.8204	1/1:0,1:1:3:40,3,0	1/1:0,2:2:6:82,6,0	/	0/1:2,1:3:31:31,0,61
28142950	28142950	T	C	intergenic	PTHRP,CCDC91	rs1267208	0.904353	0.8204	1/1:0,2:2:6:83,6,0	/	/	/
28143462	28143462	C	T	intergenic	PTHRP,CCDC91	rs251955	0.905751	0.8204	/	1/1:0,2:2:6:47,6,0	1/1:0,1:1:3:40,3,0	0/0:2,0:2:6:0,6,49
28145950	28145950	C	T	intergenic	PTHRP,CCDC91	rs251952	0.905751	0.8204	/	/	1/1:0,3:3:9:89,9,0	/
28148641	28148641	A	G	intergenic	PTHRP,CCDC91	rs805513	0.905751	0.8204	1/1:0,4:4:12:167,12,0	/	1/1:0,1:1:3:43,3,0	0/0:2,0:2:6:0,6,79
28173089	28173089	T	C	intergenic	PTHRP,CCDC91	rs7972767	0.832867	0.8681	1/1:0,2:2:6:83,6,0	1/1:0,1:1:3:42,3,0	/	/
28186208	28186208	T	C	intergenic	PTHRP,CCDC91	rs10771400	0.000599	/	0/1:5,2:7:69:69,0,251	0/0:3,0:3:9:0,9,211	0/0:1,0:1:3:0,3,83	0/0:4,0:4:12:0,12,198
28207017	28207017	A	C	intergenic	PTHRP,CCDC91	rs7970617	/	/	0/1:4,2:6:69:69,0,251	0/0:3,0:3:9:0,9,211	0/0:1,0:1:3:0,3,83	0/0:4,0:4:12:0,12,198
28208643	28208643	T	G	intergenic	PTHRP,CCDC91	rs35427724	0.413339	0.2629	1/1:0,2:2:6:84,6,0	0/0:1,0:1:3:0,3,37	0/0:1,0:1:3:0,3,38	0/0:1,0:1:3:0,3,38

28214089	28214089	T	C	intergenic	PTHRP,CCDC91	rs1554986	0.675319	0.8284	0/0:1,0:1:3:0,3,42	/	1/1:0,3:3:9:112,9,0	1/1:0,1:1:3:38,3,0
28214138	28214138	T	C	intergenic	PTHRP,CCDC91	rs1554985	0.784345	0.9226	/	/	/	1/1:0,3:3:9:104,9,0
28223555	28223555	G	C	intergenic	PTHRP,CCDC91	rs1600345	0.713658	0.9097	1/1:0,2:2:6:78,6,0	/	/	/
28224911	28224911	A	G	intergenic	PTHRP,CCDC91	rs1842198	0.713658	0.9097	1/1:0,2:2:6:80,6,0	/	/	/
28224912	28224912	T	C	intergenic	PTHRP,CCDC91	rs1842197	0.560104	0.6905	/	0/1:1,2:3:32:75,0,32	1/1:0,1:1:3:43,3,0	1/1:0,1:1:3:43,3,0
28226654	28226654	A	G	intergenic	PTHRP,CCDC91	rs438277	0.72504	0.7113	1/1:0,2:2:6:90,6,0	/	/	/
28229304	28229304	T	G	intergenic	PTHRP,CCDC91	rs533762	0.725639	0.7123	1/1:0,2:2:6:90,6,0	/	/	/
28229358	28229358	A	G	intergenic	PTHRP,CCDC91	rs493401	0.725439	0.7153	/	0/1:1,3:4:28:68,0,28	/	/
28262880	28262880	C	T	intergenic	PTHRP,CCDC91	rs2347595	0.636382	0.6915	0/1:1,1:2:31:36,0,31	/	1/1:0,2:2:6:49,6,0	1/1:0,2:2:6:49,6,0
28271656	28271656	C	T	intergenic	PTHRP,CCDC91	rs899504	0.53095	0.6438	/	0/0:1,0:1:3:0,3,33	1/1:0,2:2:6:49,6,0	1/1:0,2:2:6:49,6,0
28272574	28272574	G	A	intergenic	PTHRP,CCDC91	rs2881788	0.569289	0.6101	1/1:0,2:2:6:77,6,0	/	/	/
28280928	28280928	T	C	intergenic	PTHRP,CCDC91	rs10219630	0.524361	0.6101	1/1:0,5:5:15:127,15,0	/	/	0/0:1,0:1:3:0,3,43

28283557	28283557	C	T	intergenic	PTHRP,CCDC91	rs4931726	0.52516	0.6131	1/1:0,1:1:3:40,3,0	1/1:0,4:4:12:150,12,0	0/0:2,0:2:6:0,6,86	0/0:1,0:1:3:0,3,43
28283780	28283780	A	G	intergenic	PTHRP,CCDC91	rs7310453	0.994209	1	1/1:0,2:2:6:49,6,0	1/1:0,2:2:6:49,6,0	1/1:0,1:1:3:43,3,0	/
28299837	28299837	C	T	intergenic	PTHRP,CCDC91	rs11049370	0.572883	0.6151	1/1:0,3:3:9:89,9,0	/	/	/
28314076	28314076	T	C	intergenic	PTHRP,CCDC91	rs117785954	0.578075	0.6151	1/1:0,2:2:6:73,6,0	/	/	/
28322217	28322217	T	C	intergenic	PTHRP,CCDC91	rs10843121	0.163938	0.0446	0/1:2,2:4:65:65,0,69	0/0:10,0:10:30:0,30,357	0/0:10,0:10:30:0,30,310	0/0:9,0:9:27:0,27,259
28325997	28325997	T	C	intergenic	PTHRP,CCDC91	rs1842195	0.004393	0.0198	0/0:5,0:5:15:0,15,168	0/0:2,0:2:6:0,6,78	0/1:1,5:6:23:123,0,23	0/0:1,0:1:3:0,3,41
28336713	28336713	C	T	intergenic	PTHRP,CCDC91	rs61920228	0.557508	0.5823	1/1:0,2:2:6:66,6,0	/	0/0:1,0:1:3:0,3,38	0/0:1,0:1:3:0,3,38
27957293	27957295	TCT	-	intergenic	KLHL42,PTHRP	rs150733182	0.186701	0.2808	0/0:1,0:1:3:0,3,49	0/0:2,0:2:6:0,6,98	1/1:0,4:4:12:160,12,0	0/1:1,5:6:31:166,0,31
27963337	27963338	AC	inter-genic		KLHL42,PTHRP	/	/	/	0/0:3,0:3:9:0,9,133	0/0:2,0:2:6:0,6,90	0/0:1,0:1:3:0,3,45	0/1:2,2:4:71:71,0,78
27979758	27979763	CCTTGA	-	intergenic	KLHL42,PTHRP	rs147364420	0.961661	0.999	1/1:0,2:2:6:90,6,0	1/1:0,3:3:9:135,9,0	/	/
28031280	28031280	-	ACACAC	intergenic	KLHL42,PTHRP	/	/	/	/	1/1:0,2:2:6:90,6,0	/	/
28050688	28050688	C	-	intergenic	KLHL42,PTHRP	rs398019011	1	1	1/1:0,2:2:6:75,6,0	1/1:0,1:1:3:37,3,0	1/1:0,2:2:6:75,6,0	/

28052501	28052501	-	TGTGTG	intergenic	KLHL42,PTHRP	rs71438720	/	/	/	1/1:0,2:2:6:90,6,0	/	/
28063329	28063329	-	T	intergenic	KLHL42,PTHRP	rs397823807	1	1	/	1/1:0,2:2:6:75,6,0	1/1:0,2:2:6:75,6,0	1/1:0,2:2:6:75,6,0
28072079	28072079	-	G	intergenic	KLHL42,PTHRP	rs71039862	0.893371	0.875	/	1/1:0,1:1:3:40,3,0	1/1:0,3:3:9:121,9,0	1/1:0,1:1:3:40,3,0
28108862	28108862	G	-	intergenic	KLHL42,PTHRP	/	/	/	/	/	1/1:0,2:2:6:75,6,0	/
28111319	28111319	-	A	UTR3	PTHRP	rs34525777	0.597045	0.5427	1/1:0,17:17:51:425,51,0	0/1:4,5:9:69:100,0,69	0/1:9,14:23:99:302,0,143	0/0:11,0:11:32:0,32,240
28115165	28115165	-	TAGA	intronic	PTHRP	rs35742272	0.278954	0.2361	1/1:0,2:2:6:90,6,0	/	/	/
/28117192	28117192	T	-	intronic	PTHRP	rs369112302	/	/	/	/	1/1:0,3:3:9:135,9,0	/
28117199	28117201	CTC	-	intronic	PTHRP	rs374738849	/	/	/	/	1/1:0,3:3:9:135,9,0	/
28117405	28117406	AG	-	intronic	PTHRP	rs10551155	0.750998	0.7331	1/1:0,2:2:6:70,6,0	1/1:0,4:4:12:136,12,0	1/1:0,2:2:6:70,6,0	/
28122657	28122662	CACAGC	-	UTR5	PTHRP	rs148094002	0.022165	0.0724	0/0:84,0:84:99:0,253,5207	0/0:57,0:57:99:0,171,3834	0/0:112,0:112:99:0,336,7227	0/1:33,18:51:99:657,0,2002
28140227	28140227	A	-	intergenic	PTHRP,CCDC91	rs34784016	0.907149	0.8254	1/1:0,1:1:3:25,3,0	1/1:0,4:4:12:101,12,0	/	0/0:3,0:3:9:0,9,84

28143392	28143393	AA	-	intergenic	PTHRP,CCDC91	rs138562450	0.889776	0.8204	/	1/1:0,3:3:9:101,9,0	1/1:0,1:1:3:20,3,0	0/0:2,0:2:6:0,6,39
28207974	28207974	-	TC	intergenic	PTHRP,CCDC91	rs4035312	0.864816	0.9742	/	1/1:0,5:5:15:177,15,0	1/1:0,2:2:6:72,6,0	/
28208636	28208637	AT	-	intergenic	PTHRP,CCDC91	rs139334326	/	/	/	/	/	1/1:0,2:2:6:79,6,0
28280980	28280980	C	-	intergenic	PTHRP,CCDC91	rs58708720	1	1	1/1:0,2:2:6:71,6,0	1/1:0,1:1:3:36,3,0	1/1:0,1:1:3:36,3,0	/
28282587	28282587	-	AAG	intergenic	PTHRP,CCDC91	rs10645250	0.577476	0.6141	1/1:0,1:1:3:45,3,0	1/1:0,2:2:6:90,6,0	/	/
28336969	28336969	-	AAATGCAAAAAGAAATGAA	intergenic	PTHRP,CCDC91	rs55823056	/	/	0/1:12,9:21:99:315,0,1790	0/1:9,6:15:99:218,0,1100	0/0:19,0:19:60:0,60,2655	0/0:5,0:5:18:0,18,698

MYL3: myosin light chain 3； PTH1R: parathyroid hormone receptor 1; CCDC12: coiled-coil domain-containing protein 12; BTBD10: BTB/POZ domain-containing protein 10; PTH: parathyroid hormone; FAR1: fatty acyl-CoA reductase 1; KLHL42: kelch like family member 42; PTHRP: parathyroid hormone-related protein preproprotein; CCDC91: coiled-coil comain containing 91.

Supplementary Table S3 Quality control and alignment analysis of WES data.

Sample	Total reads	Average sequencing depth	Q20 %	Q30 %	Mapping rate on genome (%)	Coverage of exome (%)	Fraction of target covered ≥ 4x（%）	Fraction of target covered ≥ 20x (%)
IV-1	64389804	77.95	97.01	92.1	99.86	99.23%	99.04%	94.96%
III-1	70417150	85.89	96.68	91.43	99.82	99.47%	99.31%	95.92%

Supplementary Table S4. Calling analysis of WES data.

SNPs	Exonic	Intronic	Missense	Indels	Frameshift deletion	Frameshift insertion	Stoploss	Stopgain
287138	21959	140854	10140	55880	164	143	7	81

WES: Whole exome sequencing; SNP: single nucleotide polymorphism.

Supplementary Table S5 Training genes for ToppGene analysis

	Entrez Gene ID	Gene Symbol	Gene name
1	7043	TGFB3	transforming growth factor beta 3
2	7046	TGFBR1	transforming growth factor beta receptor 1
3	4487	MSX1	msh homeobox 1
4	4488	MSX2	msh homeobox 2
5	2248	FGF3	fibroblast growth factor 3
6	7049	TGFBR3	transforming growth factor beta receptor 3
7	2249	FGF4	fibroblast growth factor 4
8	650	BMP2	bone morphogenetic protein 2
9	652	BMP4	bone morphogenetic protein 4
10	2253	FGF8	fibroblast growth factor 8
11	2254	FGF9	fibroblast growth factor 9
12	3664	IRF6	interferon regulatory 6

13	1745	DLX1	distal-less homeobox 1
14	1746	DLX2	distal-less homeobox 2
15	23314	SATB2	SATB homeobox 2
16	2260	FGFR1	fibroblast growth factor receptor 1
17	8085	KMT2D	lysine methyltransferase 2D
18	5083	PAX9	paired box 9
19	84634	KISS1R	KISS1 receptor
20	860	RUNX2	RUNX family transcription factor 2
21	5727	PTCH1	patched 1
22	54880	BCOR	BCL6 corepressor
23	8481	OFD1	OFD1 centriole and centriolar satellite protein
24	26468	LHX6	LIM homeobox 6
25	999	CDH1	cadherin 1
26	3688	ITGB1	integrin subunit beta 1
27	7403	KDM6A	lysine demethylase 6A

28	1005	CDH7	cadherin 7
29	7473	WNT3	Wnt family member 3
30	8626	TP63	tumor protein p63
31	7474	WNT5A	Wnt family member 5A
32	8313	AXIN2	axin 2
33	5818	NECTIN1	nectin cell adhesion molecule 1
34	7291	TWIST1	twist family bHLH transcription factor 1
35	5308	PITX2	paired like homeodomain 2
36	7039	TGFA	transforming growth factor alpha
37	9608	PTH1R	parathyroid hormone 1 receptor
38	13726	KMT2C	lysine methyltransferase 2D
39	1277	COL1A1	collagen type I alpha 1
40	256764	WDR72	WD repeat domain 72
41	4054	LTBP3	latent transforming growth factor beta binding protein 3
42	54757	FAM20A	FAM20A golgi associated secretory pathway pseudokinase

Supplementary Table S6 Test results for ToppGene analysis

Rank	Gene Symbol	Gene ID	Averrage score	Overall *P* value
1	TMEM119	338773	0.591441	0.006652
2	PCSK4	54760	0.474962	0.020284
3	FAM83G	644815	0.298837	0.094564

The overall *P* value < 0.01 was taken to represent a significant difference.

Tmem119: transmembrane protein 119; PCSK4: proprotein convertase subtilisin/kexin type 4; FAM83G: family with sequence similarity 83 member G.

Supplementary Table S7 Primer pairs used in RT-qPCR analysis

Gene	Sense primer (5’-3’)	Antisense primer (5’-3’)
Tmem119	ATCACGTACTGGCGGAAGAA	GAGTTCCGCCATGACTTCTCT
Runx2	AGGTCGGTGTGAACGGATTTG	TGTAGACCATGTAGTTGAGGTCA
Ocn	TACACGTGCAGGTCAATCCC	GTTTGGCTTTAGGGCAGCAC
Osx	TGCTATACTCTGGGGGCTCT	ACAAAGCTCAGGGGGAATCG
Bsp2	CAGGGAGGCAGTGACTCTTC	AGTGTGGAAAGTGTGGAGTT
Col1α1	ATGCCGCGACCTCAAGATG	TGAGGCACAGACGGCTGAGTA
Alp	CCAACTCTTTTGTGCCAGAGA	GGCTACATTGGTGTTGAGCTTTT
Atf4	TGCCAGATGAGCTCTTGACC	TCATAAGGTTTGGGCCGAGG
Glut1	GTAAGCGTTGTCCAGGGTGA	TGCATTGCCCATGATGGAGT
G6pd	TGGAGGAGTTCTTTGCCCGTAATTC	GTGGCTGTGAGGTGCTTATAGGAG
Ldha	GCTCCCCAGAACAAGATTACAG	TCGCCCTTGAGTTTGTCTTC
Pkm2	TCGCCCTTGAGTTTGTCTTC	GTAAGCGTTGTCCAGGGTGA

Hk1	CGAGCTGAAGGATGACCAAGT	TGATCAGGCCGACTTCACAC
Pgk1	GAGCCTCACTGTCCAAACTAGG	TCTGCTTAGCTCGACCCACA
Gapdh	AGGTCGGTGTGAACGGATTTG	TGTAGACCATGTAGTTGAGGTCA
β-Actin	GTTGGAGCAAACATCCCCCA	CGCGACCATCCTCCTCTTAG

Tmem119: transmembrane protein 119; Runx2: RUNX family transcription factor 2; Osx: Osterix; Ocn: osteocalcin; Bsp2: bone sialoprotein II; Col1α1: collagen type I alpha 1 chain; Alp: alkaline phosphatase; Atf4: activating transcription factor 4; Glut1: glucose transporter type 1; G6pd: glucose-6-phosphate dehydrogenase; Ldha: lactate dehydrogenase A; Pkm2: pyruvate Kinase M2; Hk1: hexokinase 1; Pgk1: phosphoglycerate kinase 1; Gapdh： glyceraldehyde-3-phosphate dehydrogenase； β-Actin： actin beta.

FIGURES

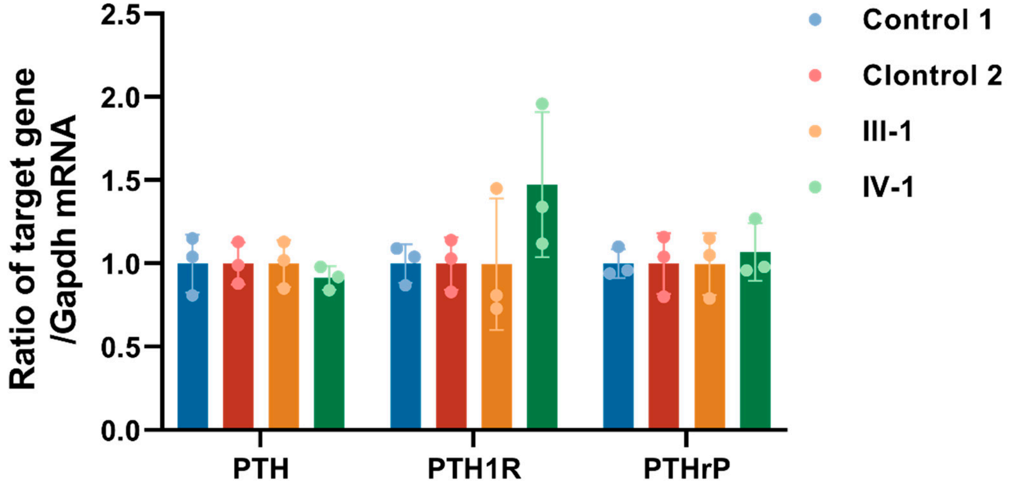

Supplementary Figure S1 The expression level of PTH, PTH1R and PTHrP gene was similar among family members. PTH: parathyroid hormone; PTH1R: Parathyroid hormone/parathyroid hormone-related peptide receptor; PTHrP: parathyroid hormone-related peptide.

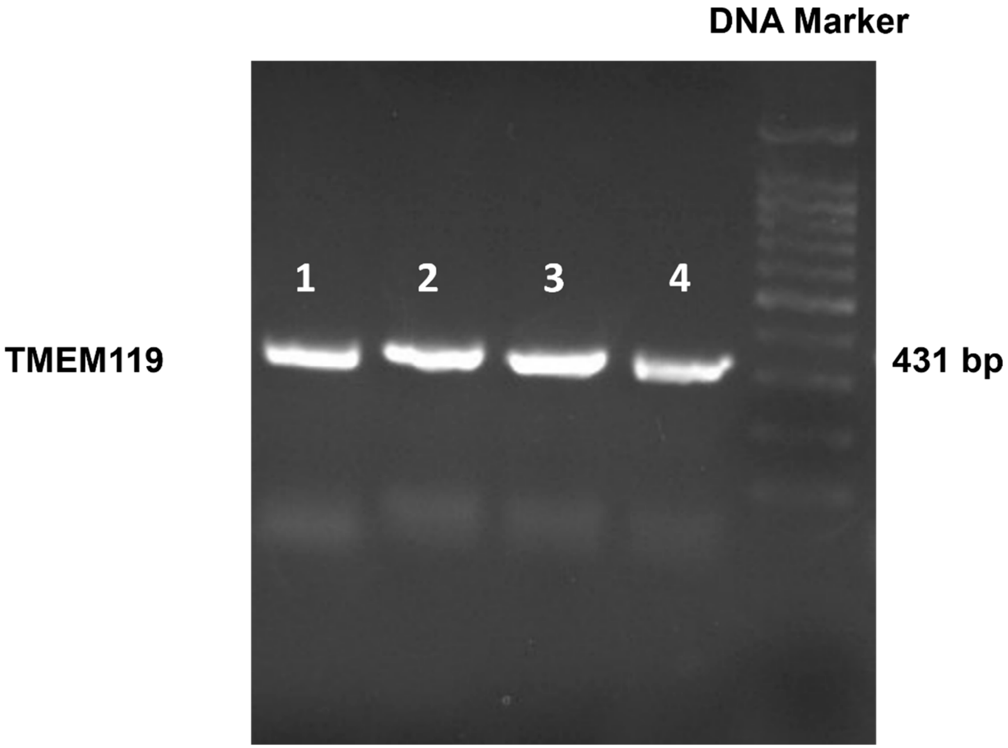

Supplementary Figure S2 PCR products were evaluated by agarose gel electrophoresis. The DNA molecules in all four samples were the same 431 bp. PCR: polymerase chain reaction; bp: base pair; TMEM119: transmembrane protein 119; bp: base pair.

Lane 1: control 1

Lane 2: control 2

Lane 3: III-1

Lane 4: IV-1

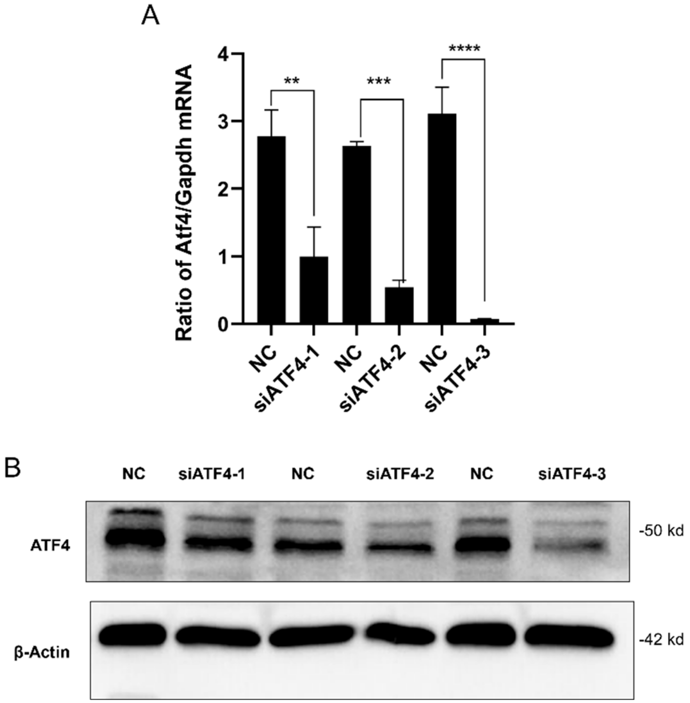

Supplementary Figure S3 Validation of the effect of ATF4 gene knockdown. **(A)** The mRNA level of ATF4 was knocked down at 48 h after siRNA transfection. **(B)** The protein level of ATF4 was knocked down at 48 h after siRNA transfection. ATF4: activating transcription factor 4; siNC: small interfering RNA-negative control; WT: wild-type; EV: empty vector. *P* values were significant at $^{**}p < 0.01$

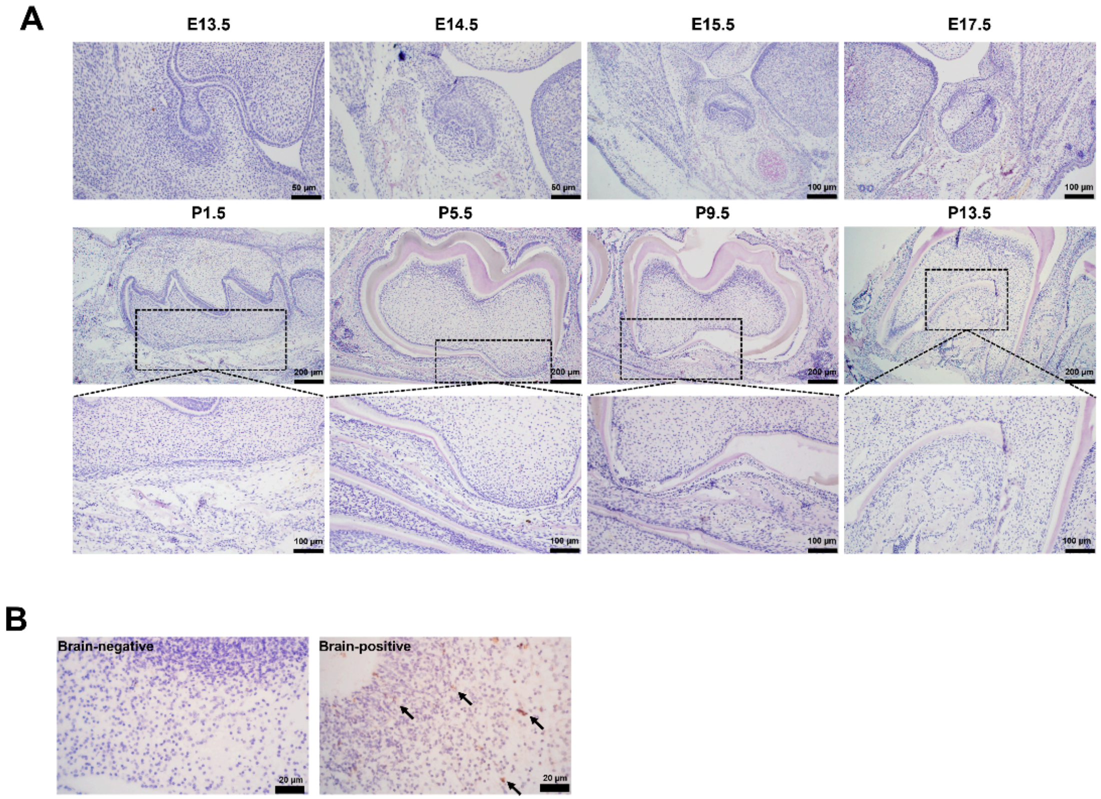

Supplementary Figure S4 Positive and negative control of Immunohistochemical staining. **(A)** Negative control of immunohistochemical staining of mice tooth germs at different developmental stages. **(B)** Negative and positive control of immunohistochemical staining of mouse brain tissue. Microglia with Tmem119-positive staining (black arrows). P: postnatal; E: embryo.
